# Evolving a terminal deoxynucleotidyl transferase for commercial enzymatic DNA synthesis

**DOI:** 10.1093/nar/gkaf115

**Published:** 2025-02-21

**Authors:** Stephanie M Forget, Mikayla J Krawczyk, Anders M Knight, Charlene Ching, Rachelle A Copeland, Niusha Mahmoodi, Melissa A Mayo, James Nguyen, Amanda Tan, Mathew Miller, Jonathan Vroom, Stefan Lutz

**Affiliations:** Codexis Inc., 200 Penobscot Drive, Redwood City, CA 94063, United States; Codexis Inc., 200 Penobscot Drive, Redwood City, CA 94063, United States; Codexis Inc., 200 Penobscot Drive, Redwood City, CA 94063, United States; Codexis Inc., 200 Penobscot Drive, Redwood City, CA 94063, United States; Codexis Inc., 200 Penobscot Drive, Redwood City, CA 94063, United States; Codexis Inc., 200 Penobscot Drive, Redwood City, CA 94063, United States; Codexis Inc., 200 Penobscot Drive, Redwood City, CA 94063, United States; Codexis Inc., 200 Penobscot Drive, Redwood City, CA 94063, United States; Codexis Inc., 200 Penobscot Drive, Redwood City, CA 94063, United States; Codexis Inc., 200 Penobscot Drive, Redwood City, CA 94063, United States; Codexis Inc., 200 Penobscot Drive, Redwood City, CA 94063, United States; Codexis Inc., 200 Penobscot Drive, Redwood City, CA 94063, United States

## Abstract

Enzymatic DNA synthesis, using stepwise nucleotide addition catalyzed by template-independent polymerases, promises higher efficiency, quality, and sustainability than today’s industry-standard phosphoramidite-based processes. We report the directed evolution of a terminal deoxynucleotidyl transferase that uses 3′-phosphate-blocked 2′-deoxynucleoside triphosphates (dNTPs) to control the polymerization reaction. Over 32 iterative rounds of laboratory evolution, 80 amino acid substitutions—constituting ∼20% of the coding protein sequence—were introduced. The engineered polymerase exhibits uniformly high catalytic activity, raising incorporation efficiency by 200-fold to >99% for dNTPs with a 3′-reversible terminator while reducing extension times by >600-fold to 90 s. The same enzyme variant displays improved enzyme robustness, as reflected in the 20°C increase in thermostability. Based on these performance characteristics, the engineered polymerase represents an operational prototype for biocatalytic DNA synthesis at a commercial scale.

## Graphical Abstract

**Graphical Abstract:**
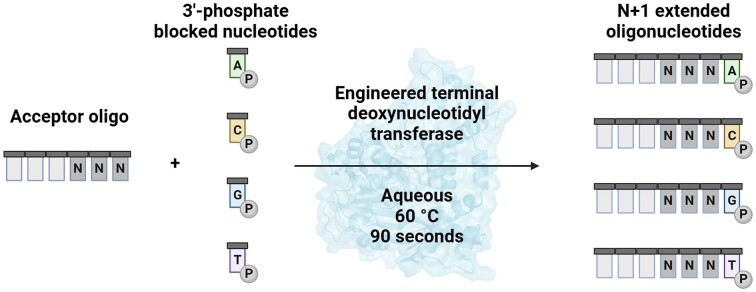

## Introduction

DNA synthesis is a cornerstone of biological research. From the assembly of new synthetic DNA constructs to the amplification or mutagenesis of existing sequences with oligonucleotide primers, research depends on the ability to write DNA accurately, quickly, and inexpensively. These constructs and primers are primarily synthesized using phosphoramidite-based chemistry first reported in 1981 [1]. Steady improvements and optimization of the process over the last four decades have driven up the average nucleotide coupling efficiency of this solid-phase synthesis to ≥99.5% [2]. Despite such superb performance, the yield of full-length DNA falls off as oligonucleotide length exceeds 150–200 base pairs, and DNA quality is diminished by repeated exposure to harsh chemical conditions during the iterative synthesis. Furthermore, phosphoramidite synthesis has poor atom economy and generates significant hazardous organic waste [3]. In contrast, enzymatic DNA synthesis can exploit the remarkable fidelity and efficiency of nature’s replication machinery while also offering a greener process that runs in aqueous conditions and minimizes the need for activators, oxidants, and protection groups.

Conceptually, enzymatic DNA synthesis of oligonucleotides is an iterative, two-step process of extension and deblocking. In the extension step, a polymerase catalyzes the template-independent addition of a 2′-deoxynucleoside triphosphate (dNTP) to the 3′-end of a polynucleotide strand. One strategy for limiting extension to a single nucleotide involves using polymerase–dNTP conjugates to sterically hinder the 3′ terminus of the extinction product [4, 5]. Alternatively, run-away polymerization can be avoided via reversible blocking of the dNTP’s 3′-hydroxyl group, which gives the added benefit of producing scarless oligonucleotide products [6]. In the deblocking step, hydrolysis liberates the 3′-position of the growing oligonucleotide and readies it for the next cycle of nucleotide addition. High speed, coupling efficiency, and uniformity of each reaction step are critical for the translation of this concept into a competitive commercial DNA synthesis process—goals most elegantly accomplished by an all-enzymatic approach.

Terminal deoxynucleotidyl transferases (TdTs) are the most well-studied polymerases in the context of template-independent synthesis. TdT was proposed as a catalyst for enzymatic DNA synthesis using nucleotides with blocked 3′-hydroxyl groups in 1962 [7], and it has been a leading candidate for DNA synthesis applications ever since [8–11]. Steric constraints in the TdT active site have in the past favored modifications with a small steric footprint such as nitrobenzyl [12] or aminoalkoxyl [13, 14] groups. However, the removal of these entities requires photolytic or chemical unblocking/deprotection step which adds complexity to the overall process. Alternatively, a phosphate group is an effective blocking group [15] that can efficiently be hydrolyzed by commercially available phosphatases under reaction conditions similar to the TdT-catalyzed nucleotide extension step [16, 17]. These deblocking reactions can be carried out at physiological pH conditions that minimize depurination and other potential DNA damage. While 3′-phosphate blocked dNTPs (3′P-dNTPs) would enable an all-enzymatic solution to DNA synthesis, this large, polar blocking group is poorly tolerated by wild-type (WT) TdTs.

**Figure 1.**
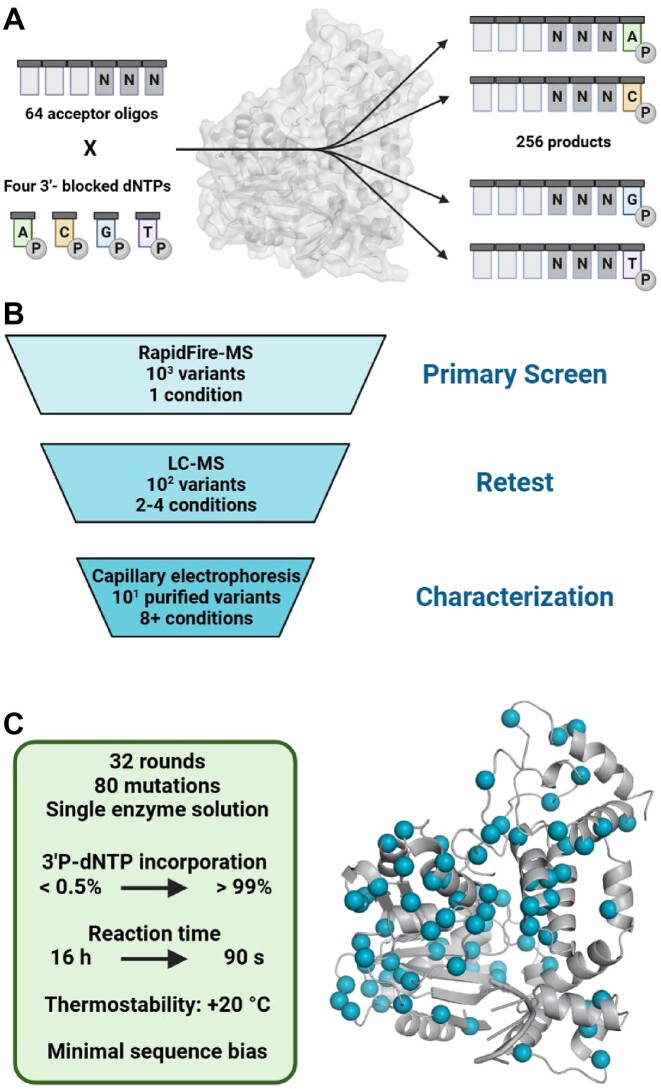
DNA synthesis problem and the enzyme engineering solution. (**A**) Combinatorial complexity of oligo synthesis with oligo sequence bias. Accounting for the three 3′-terminal nucleotides in an oligo acceptor and the four 3′P-dNTPs, an enzyme for DNA synthesis needs to catalyze 256 polymerization reactions with non-native substrates at a minimum of 99% coupling efficiency to be commercially relevant. (**B**) Multiple tiered approach to screening. A round of evolution typically consists of 500–2000 variants, and thousands of variants can be screened in one day under a single reaction condition using a RapidFire-MS system. Variants (100–400) showing improvement under the primary screen are tested under multiple additional challenge conditions (e.g. different oligo acceptors, thermostability, or dNTP equivalents). Top variants from the secondary screening are shake-flask purified and characterized under several challenge conditions, with one variant selected as the backbone for the next round of evolution. (**C**) Mutations in **TdT-33** mapped onto a homology model based on PDB ID: 4I279. Eighty mutations across 32 rounds of evolution (Supplementary Tables S9 and S10) brought the engineered **TdT-33** to high coupling efficiency and low sequence bias in 90-s reactions (Created with BioRender.com).

Protein engineering by directed evolution can be applied to address the functional impediment of TdT toward use of 3′P-dNTPs. At the same time, engineering efforts must select TdT variants with improvements to several traits required for high performance in DNA synthesis. Specifically, process requirements call for nucleotide incorporation efficiencies of >99% at reaction times of <90 s per cycle and tolerance of elevated reaction temperatures (60°C) to minimize interference of 3′-terminal DNA secondary structure with TdT binding [18]. In a departure from traditional enzyme engineering focused on tailoring one biocatalyst to one specific substrate [19–21], TdT evolution must strive for maximum substrate promiscuity. Native TdTs exhibit significant bias with respect to dNTPs and the three nucleotides at the 3′-terminus of the oligo acceptor sequence [22, 23]. Given 64 (4^3^) unique combinations of oligonucleotide 3′-terminal sequences and four 3′P-dNTP substrates, the ideal polymerase for enzymatic DNA synthesis must achieve high and uniform nucleotide coupling efficiency on 256 substrate pairs (Fig. 1A). Herein, we describe the directed evolution of TdT for use in commercial enzymatic DNA synthesis.

## Materials and methods

### Reagents

Reagents and buffers were ordered from Thermo Fisher Scientific (Waltham, MA) at molecular biology grade and used without further purification. Nucleotides were received from Molecular Assemblies, Inc. and can also be purchased from Hongene Biotech (Union City, CA). Oligonucleotides were ordered from Integrated DNA Technologies (Coralville, IA) and used without further purification. Protein was purified with ÄKTA pure™ protein purification system (Cytiva, Marlborough, MA) with 5 ml HisTrap FF columns.

### Expression of full-length and truncated wild-type TdT variants

The coding sequences of multiple WT TdT enzymes were codon optimized for expression in *Escherichia coli* and synthesized. The synthetic constructs were subcloned in *E. coli* expression vector pCK100900i, with the full-length coding sequence truncated such that the first amino acid after the N-terminal 6xHis tag corresponded to residue 132 of the *Mus musculus* TdT. The N-terminal region serves a regulatory purpose and is not necessary for *in vitro* polymerase activity [24]. TdT constructs were transformed into an *E. coli* strain derived from W3110 and grown at shake-flask scale, lysed, purified, and dialyzed into storage buffer (20 mM Tris–HCl, pH 7.4, 100 mM KCl, 0.1 mM EDTA, and 50% glycerol). After overnight dialysis, protein samples were removed and TdT concentrations were measured by absorption at 280 nm (Supplementary Table S1).

### Shake flask expression of TdT variants

Selected variants were plated onto LB agar plates with 1% glucose and 30 μg/ml chloramphenicol and grown overnight at 37°C. A single colony from each was transferred to 5 ml of LB broth with 1% glucose and 30 μg/ml chloramphenicol. The cultures were grown for 20 h at 30°C, 250 rpm, and subcultured at a dilution of ∼1:50 into 250 ml of Terrific Broth with 30 μg/ml of chloramphenicol to a final OD_600_ of ∼0.05. The cultures were incubated at 30°C, 250 rpm to an OD_600_ of ∼0.6, and then induced with Isopropyl β-d-1-thiogalactopyranoside (IPTG) at a final concentration of 1 mM. Following incubation for 20 h at 30°C, 250 rpm, the cultures were centrifuged at 4000 rpm for 10 min. The culture supernatant was discarded, and the pellets resuspended in 35 ml of 20 mM triethanolamine, pH 7.5. This cell suspension was lysed using a Microfluidizer cell disruptor (Microfluidics M‐110L) on ice. The crude lysate was pelleted by centrifugation (11 000 rpm for 60 min at 4°C), and the supernatant was filtered through a 0.2-μm polyethersulfone (PES) membrane to further clarify the lysate. For further purification, TdT lysates were supplemented with 1 ml of elution buffer [50 mM Tris–HCl, 500 mM NaCl, 250 mM imidazole, and 0.02% (v/v) Triton X-100 reagent] and run over a 5 ml HisTrap FF column (GE Healthcare) using the AC Step HiF setting on the Akta purification system (for run parameters please see Supplementary Table S2). The wash buffer comprised 50 mM Tris–HCl, 300 mM NaCl, 20 mM imidazole, and 0.02% (v/v) Triton X-100 reagent. Protein elution was monitored by UV absorption (A280) and product fractions pooled and dialyzed overnight in dialysis buffer (20 mM Tris–HCl, pH 7.4, 100 mM KCl, 0.1 mM EDTA, and 50% glycerol) in a 3.5K Slide-A-Lyzer™ dialysis cassette (Thermo Fisher) for buffer exchange. TdT concentrations were measured by absorption at 280 nm.

### Terminal deoxynucleotidyl transferase gene HTP library construction

The WT TdT enzyme is a predicted splice variant encoded by the genome of species *Empidonax traillii*. Combinatorial libraries composed of variants with one or more single amino acid substitutions were generated by using standard quick-change mutagenesis techniques. Saturation-mutagenesis libraries composed of variants with a single programmed amino acid substitution were generated with polymerase chain reaction (PCR) amplification utilizing degenerate oligos (i.e. NNK, NNN, and NNS) to incorporate mutations at the targeted position.

### High-throughput growth of TdT enzyme and variants

Libraries were transformed into an *E. coli* strain derived from W3110 and plated onto LB agar plates containing 1% glucose and 30 μg/ml chloramphenicol. After overnight incubation at 37°C, colonies were placed into the wells of 96-well shallow flat bottom NUNC™ (Thermo-Scientific) plates filled with 180 μl/well LB medium supplemented with 1% glucose and 30 μg/ml chloramphenicol. The cultures were allowed to grow at 200 rpm and 30°C. Overnight growth samples (20 μl) were transferred into Costar 96-well deep plates filled with 380 μl of Terrific Broth supplemented with 30 μg/ml chloramphenicol. The plates were incubated for 2 h at 250 rpm, 30°C until the OD_600_ reached 0.4–0.8, followed by induction with 40 μl of 10 mM IPTG in sterile water and overnight incubation at 250 rpm, 30°C. The cells were pelleted (4000 rpm for 20 min), the supernatants were discarded, and the cells were frozen at −80°C prior to analysis.

### Preparation of crude cell lysates and purified samples of TdT variants for HTP screening

Cell pellets were resuspended in 300–400 μl lysis buffer containing 50 mM Tris buffer, pH 8.0, 0.2 g/l lysozyme, and 0–300 mM NaCl and incubated at room temperature for 2 h with shaking on a bench top shaker. Following centrifugation for 15 min at 4000 rpm and 4°C, the clear supernatants were heat treated (1 h, 40–68°C depending on the round) and used directly in biocatalytic reactions to determine activity levels of crude lysate. Alternatively, TdT variants were purified from crude *E. coli* extracts by metal-affinity chromatography using HIS-Select^®^ High Capacity (HC) Nickel Coated Plates (Sigma) according to the manufacturer’s instructions. HIS-Select plates were equilibrated with a total of 800 μl of wash buffer [50 mM Tris–HCl, 300 mM NaCl, 20 mM imidazole, and 0.02% (v/v) Triton X-100 reagent] per well. Then, 200 μl of HTP lysate was loaded onto the plate, and centrifuged for 1 min at 2000 rpm, 4°C. The plate was washed twice with 400 μl of wash buffer/well, followed by centrifugation for 3 min at 3000 rpm, 4°C. TdT samples were eluted with the addition of 100 μl elution buffer [50 mM Tris–HCl, 300 mM NaCl, 350 mM imidazole, and 0.02% (v/v) Triton X-100 reagent] by centrifugation for 1 min at 3000 rpm, 4°C. Eluates were buffer-exchanged into TdT storage buffer (20 mM Tris–HCl, pH 7.5, 100 mM KCl, 0.1 mM EDTA, and 50% glycerol) using Zeba™ Spin desalting plates (Thermo Fisher).

### Biochemical characterization of TdT variants in HTP screening assay

In 200 μl of Bio-Rad PCR plates, reactions included 1–4 μM oligonucleotide, 5–200 mM nucleotide triphosphate, 0.002 Unit/μl yeast pyrophosphatase (Thermo Scientific), 20 mM buffer, 50 mM potassium acetate, 25 vol% TdT (lysate or purified sample), and 250 μM cobalt (II) chloride. All reaction components, except for TdT, were premixed in a single solution, and 15 μl of this solution was aliquoted into each well of the 96-well plates. Upon addition of 5 μl of TdT solution to each well to initiate the reaction, the plate was heat-sealed with a peelable aluminum seal and incubated in a thermocycler at the indicated temperature and reaction time, and then held at 4°C. For analysis by the LC-MS, reactions were quenched by the addition of 24 μl of acetonitrile and 16 μl of 20 mM aqueous EDTA and characterized by the LC-MS using either method A (Agilent Ultivo) or method B (Thermo LTQ XL) (see Supplementary Tables S3 and S4). For analysis by RapidFire, reactions were quenched by adding 40 μl of acetonitrile/methanol 9:1 and clarifying by centrifugation. The supernatants were diluted with one volume of water and transferred to 384-well plates for analysis on the Agilent RapidFire SPE-MS/MS (Supplementary Table S5).

### Biochemical characterization of selected TdT variants

Reactions contained 3′phos-dNTP (2.5–320 μM), 1 μM oligo, TdT variant (1–2 μM), 0.01 U *E. coli* IPP (NEB), 20 mM 3-(N-morpholino)propanesulfonic acid (MOPS) (pH 7.6), 50 mM KOAc, 0.25 mM CoCl_2_, 5% formamide, and 0.625% PEG-3350. The 3′P-dNTP concentration, TdT variant concentration, and preincubation temperatures varied per experiment and are defined in the corresponding figure legends. Reaction plates and solutions were kept cold during setup. To set up reactions, a 5-μl TdT solution was added to 15 μl of a solution containing all other reaction components in a 96-well skirted PCR plate (Bio-Rad). The plate was then vortexed and centrifuged at 4°C before being transferred to a thermocycler that was preheated to the reaction temperature. After a 90-s incubation, the plate was heated at 95°C for 2 min to deactivate the enzyme. The quenched reactions were subsequently diluted in the preparation for CE analysis. Reactions were initiated by the addition of 5 μl of TdT solution to 15 μl of all other reaction components in a 96-well skirted PCR plate (Bio-Rad). Plates and solutions were kept cold prior and during initiation. Components were mixed and centrifuged prior to transfer to a thermal cycler that was preheated to 45°C. The plates were preincubated for 90 s, and then heated at 95°C for 2 min. Following heat-kill, reactions were further diluted in preparation for analysis using the CE instrument.

### RapidFire SPE-MS/MS analysis of oligonucleotides

Typical reaction samples contain 4 μM TTTTTTTATC (substrate), 50 μM 3′P-dTTP, 1× MOPS buffer, pH 7.2, 0.002 U inorganic pyrophosphatase, 0.25 mM CoCl_2_, and purified TdT polymerase (positive sample) or equivalent amount of solvent (negative sample). After incubation, samples were quenched with two volumes of a mixture of 90% acetonitrile and 10% methanol and centrifuged at 4000 rpm for 10 min. Supernatants of positive and negative samples were injected on to the HILIC cartridge in alternating pattern. Various solid phase extraction conditions were tested (Supplementary Table S6). Final improved Rapidfire conditions for TTTTTTTATC are listed in Supplementary Table S7.

For method validation, a duplicate set of TdT library reaction plates producing TTTTTTTATCG3′P was generated. One set was quenched according to the RapidFire method, and another was quenched according to the HPLC MS/MS Ultivo method. The correlation value between the RapidFire and HPLC MS/MS Thermo was 0.82.

For library screening, reactions were quenched as described for the Rapid HTP screening protocol. Quenched and clarified reaction supernatant (70 μl) was then transferred to a 384-well microtiter PCR plate for MS analysis. Product was detected by RapidFire SPE-MS/MS with the instrument and parameters provided in Supplementary Table S8.

### Analysis of oligonucleotides and TdT reactions by capillary electrophoresis

For analysis of the reaction samples, capillary electrophoresis (CE) was performed using an ABI 3500xl Genetic Analyzer (Thermo Fisher). Quenched reactions were diluted 1:200 in nuclease-free water, and then diluted 1:10 in Hi-Di™ Formamide (Thermo Fisher) containing an appropriate size standard (LIZ or Alexa633). The ABI3500xl was configured with POP6 polymer, 50 cm capillaries, and a 45°C oven temperature. Pre-run settings were 18 kV for 180 s. Injection was 5 kV for 5 s, and the run settings were 19.5 kV for 600 s. Oligonucleotide substrates with 5′-FAM fluorescent label and products were identified by their sizes relative to the sizing ladder, with substrate oligos peaks at 20 bp and 3′-phosphorylated single-nucleotide extension products appearing in the region of ∼16–17 bp.

## Results and discussion

The N-terminally truncated TdT from *Empidonax traillii* was selected from a panel of WT TdTs as the evolution parent. This variant was selected due to its modest heterologous expression in *E. coli* (Supplementary Table S1) and its distance in sequence space from well-characterized members of the TdT family. The sequence was N-terminally truncated to the catalytic domain. This selected evolution parent (**TdT-01**) had trace starting activity with 3′P-dNTPs (0.25% conversion in 16 h and Supplementary Fig. S1) and required significant improvements to stability, solubility, and expression to become a viable commercial catalyst.

Evolution in the initial rounds focused on improving soluble expression and stability (Supplementary Fig. S2). To select for these traits, libraries were screened with a thermal challenge using 2′,3′-dideoxynucleotide triphosphates (ddNTPs) as surrogate substrates. Heat-treated, clarified *E. coli* lysates were used as the source of TdT for screening. Crude *E. coli* lysates contain oligonucleotide-degrading nucleases and dNTP-degrading phosphatases, which potentially complicated analysis of screening results. However, mild heat treatment was found to be sufficient to reduce these side reactions and to allow for reliable monitoring of TdT product formation. Compared to the DNA substrate, ddNTP-extended products showed higher resistance to degradation under lysate screening conditions. It was therefore possible to monitor ddNTP-extended products using LC-MS to identify beneficial variants in the context of a crude lysate screen.

After two rounds of evolution, activity on the target 3′P-dNTP substrate was measurable but could not be easily screened in lysate due to interference from lysate components. However, improvements in the stability and solubility of TdT variants over the first rounds made it possible to recover sufficient yields of TdT using a high-throughput (HTP) purification protocol. In this protocol, cell pellets were resuspended in buffer containing lysozyme then subjected to a two-step purification procedure utilizing Ni-NTA affinity followed by desalting. Screening with the purified enzymes allowed for longer incubation times (1–2 h) without product degradation, making it possible to screen for 3′P-dNTP incorporation despite low activity. At this point, HTP screening was being performed with relatively high-incorporating oligo substrates to find variants that would accommodate the 3′-phosphate blocking group.

Given the low starting activity with 3′P-dNTPs and initial evolution trajectory, we predicted that a significant number of rounds (>20) would be required to evolve TdT to meet target process requirements. Thus, we sought to simplify screening as much as possible to reduce operational complexity, time, and cost for each round of evolution. The HTP purification procedure that was initially required to screen with 3′P-dNTPs was lengthy and of limited throughput, so efforts were made to return to screening using lysates. As a result of cumulative activity and stability improvements, it became possible to screen reactions with 3′P-dNTPs using TdT in *E. coli* lysates by round 9 of evolution. Higher enzyme stability allowed for higher lysate thermal pre-treatments, which reduced degradation of oligo and 3′phosphate-dNTP by lysate components while improved activity enabled detection of 3′-phosphorylated oligo products with lower lysate loading and shorter reaction times. From this point forward, all HTP screening was with heat-treated cell lysates.

With these HTP lysate assays established, the subsequent rounds focused on increasing promiscuity, reducing bias toward oligo sequences, and improving incorporation efficiency for 3′P-dNTPs. In parallel, we continued to monitor and select for improvements in soluble expression and stability. By this point in evolution, an optimized workflow was developed (Fig. 1B). Key to enabling the evolution was the development of fast, reliable, and robust analytical methods to enable tiered HTP screening at progressively process-relevant conditions.

Primary screening represents a first pass to identify active variants using a single reaction condition. The primary screening assay relied on a Rapid-Fire MS (Agilent) analytical method using a HILIC cartridge to swiftly analyze and select improved variants, which enabled screening of over 2000 variants overnight. Active and sequence unique variants identified in the primary screen were regrown for additional testing with different screening pressures to improve reaction rate, thermal stability, or sequence bias. As part of this secondary screening, retest plates (typically 100–200 variants) were screened under 2–4 additional challenge conditions to further vet individual mutations, and lower-throughput LC-MS assays were used to track additional species of interest. TdT amino acid substitutions that produced by-products such as multiply extended or 3′-dephosphorylated products were tracked and selected against when appropriate.

**Figure 2.**
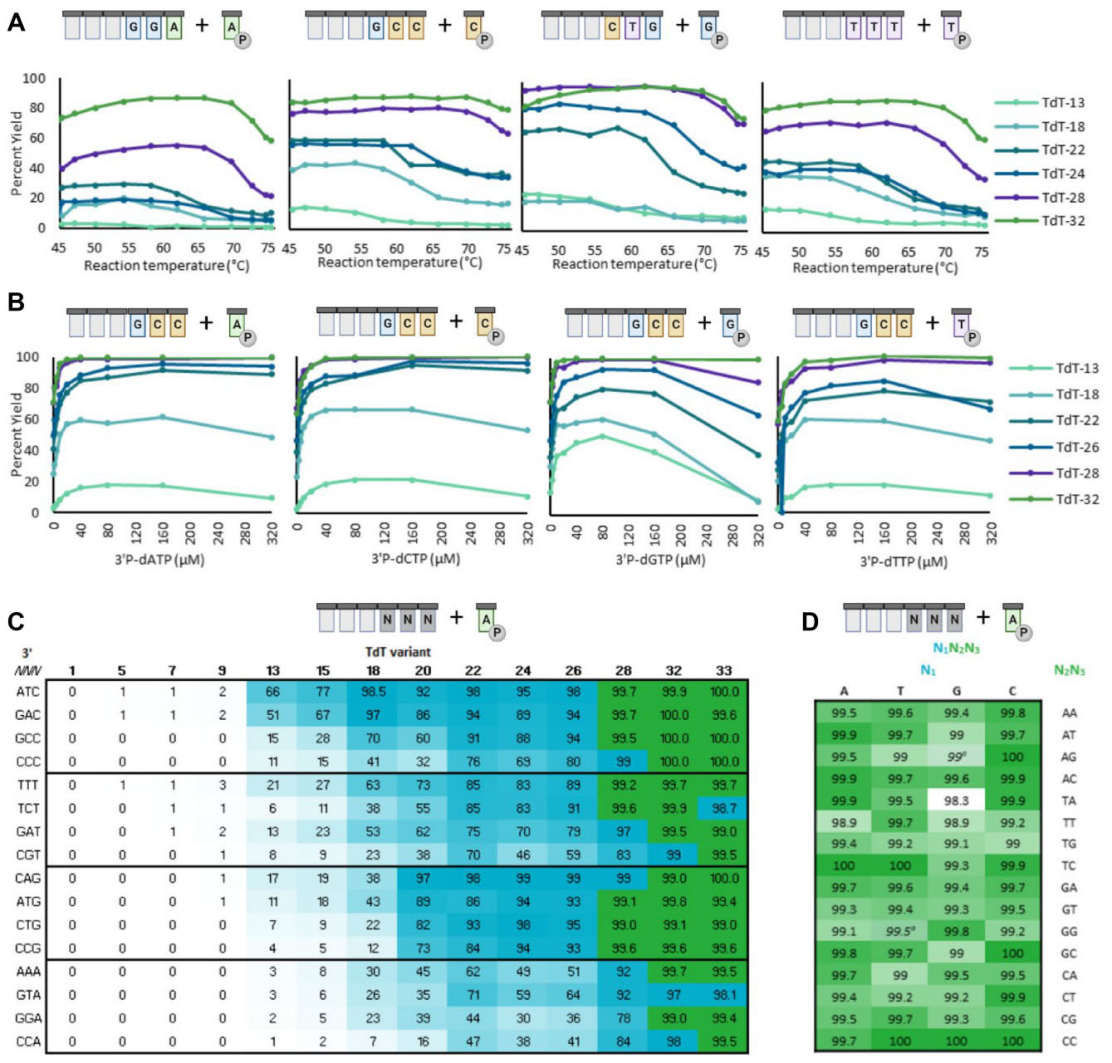
TdT performance improvements during directed evolution. (**A**) TdT variant activity at varying temperatures. Residual TdT activity following a thermal challenge from 45 to 75°C were assayed with each 3′P-dNTP and TdT variants from **TdT-13** to **TdT-32**. (**B**) Reduction in required concentration of blocked nucleotide during evolution. TdT activity was measured with varying concentrations of each 3′P-dNTP and TdT variants from **TdT-13** to **TdT-32**. (**C**) Sequence bias improvements through evolution. Nucleotide coupling efficiency with 3′P-dATP and 16 varied oligonucleotide acceptors in a 90-second reaction using 14 TdT variants from **TdT-01** to **TdT-33**. Coupling efficiencies above 99% display an additional decimal place. Reaction conditions: 2 μM TdT, 1 μM oligo, 5 μM 3′P-dATP, 90s, 60°C. (**D**) Oligo acceptor sequence bias. Coupling efficiency of **TdT-33** with 64 oligo acceptors comprising the full set of 3′-terminal N_1_N_2_N_3_ sequences was measured in a 90-s reaction. Measurements denoted with * indicate a 180-s reaction. Reaction conditions: 2 μM TdT, 1 μM oligo, 5 μM 3′P-dATP, 90s, 60°C (Created with BioRender.com).

Mutations found to be beneficial in primary and retest screening were folded into combinatorial libraries. Combinatorial libraries were evaluated by secondary screening, using 2–4 conditions and LC-MS analytical detection. A small set of variants (typically 6–8) with improved activities across multiple selection pressures were then screened in a third tier of validation assays using shake-flask-purified enzymes. These enzymes were evaluated for activity and stability improvements using either LC-MS or CE such that a detailed analysis of the reaction profile could be obtained. Mirroring our approach to HTP screening, reactions with purified proteins were evaluated using screening pressures including short reaction times, low-activity substrates, and reduced 3′P-dNTP equivalents. In each round, the variant with the best overall improvement across multiple traits was selected as the parent for the next round of evolution. Throughout evolution, variants were screened against a panel of oligo substrates bearing variable sequences at the last three 2′-deoxyribonucleotide positions at the 3′-terminus, and under-performing substrates were identified for primary and retest screening. Thirty-two rounds of evolution were completed using these approaches (conditions summarized in Supplementary Table S11). Over the course of these rounds of evolution, each position in TdT (following the N-terminal His-tag) was targeted at least once, 75% of the single amino acid substitution landscape was observed, and 80 mutations were incorporated into **TdT-33** with respect to **TdT-01** (Fig. 1C). Representative TdT variants from the evolution lineage were selected for head-to-head comparison to illustrate progress (Fig. 2).

The stability of the TdT variants improved over the course of evolution. To demonstrate this improvement, residual TdT activities after thermal challenge were assayed using ddGTP as a surrogate substrate, as early variants had minimal measurable activity on 3′P-dNTP substrates (Supplementary Fig. S3). Under these conditions, the final two variants (**TdT-32** and **TdT-33**) retained full activity up to 64°C, a 20°C improvement over the WT enzyme (**TdT-01**). Importantly, these stability gains were not achieved by steady improvements. Over the course of evolution, losses of stability were concomitantly observed with improvements in specific activity, but these losses were recoverable in subsequent rounds. For example, **TdT-22** was less thermostable than **TdT-18** but showed far less oligo acceptor sequence bias. Activity–stability trade-offs such as this underline the importance of continuously screening for the variety of traits needed in the final desired enzyme.

Variants after **TdT-13** gave measurable activity with 3′P-dNTPs and some oligonucleotides under the process-like conditions of short reaction times (90 s) and elevated temperatures (60°C). In assays measuring the activity of six TdT variants (covering **TdT-13** through **TdT-32**) at different reaction temperatures (without preincubation), round-over-round improvements with 3′P-dNTP substrates were observed alongside increases in the maximum conversion achieved (Fig. 2A). Maximum conversion is also observed at higher reaction temperatures with later variants. Continued improvement of stability alongside activity led to variants that displayed high conversion at temperatures above the 60°C desired process condition, thereby maintaining a buffer of thermostability for a robust process.

In addition to stability, 3′P-dNTP loading needed to be optimized because reagent costs are a major driver for DNA synthesis cost. While early rounds of evolution were run with 200 molar equivalents of 3′P-dNTP, the molar equivalent excess was reduced 40-fold over consecutive rounds of evolution, with **TdT-32** and **TdT-33** reaching high conversions at 5 molar equivalents 3′P-dNTPs (Fig. 2B–D). Nearly even incorporation of each 3′P-dNTP was observed with later evolution variants, although pyrimidines remained slightly more favored than purines.

A panel of 16 substrates was screened under target process conditions with 14 TdT variants selected from the evolution lineage (Fig. 2C). No activity was observed with early variants under these conditions, but by round 13, TdT variants had high activity on a few substrates, particularly oligos terminating with cytidine, and the main evolutionary pressure became decreasing sequence bias. Activity was observed to increase steadily up to **TdT-22**, at which point there were trade-offs between activity and other traits that were also being targeted for improvement, such as stability and selection against by-products. With **TdT-28**, nearly all sequences reached >90% conversion, and with **TdT-33**, reactions with all but one sequence reached 99% conversion (Fig. 2D).

## Conclusion

Variant **TdT-33** was found to meet most of the key enzyme performance targets, thereby providing proof of concept for a polymerase compatible with an all-enzymatic DNA synthesis platform. Aided by the development of efficient screening protocols and robust, fast analytical methods, **TdT-33** was developed within 14 months of the onset of the evolution campaign. Throughout evolution, TdT’s soluble expression, thermostability, 3′P-dNTP loading, and oligo acceptor sequence bias were maintained or improved. Even after 32 rounds of evolution, improvements in each desired trait continued to be observed, including activity on the most challenging substrate pairs. Additional evolution has been projected to further improve performance, and continued enzyme evolution and process development are ongoing to realize an enzymatic route to long, high-purity synthetic DNA.

## Supplementary Material

gkaf115_Supplemental_File

## Data Availability

The data underlying this article are available in the article and in its online Supplementary Data.
