# Supplementary material for: Evolving a terminal deoxynucleotidyl transferase for commercial enzymatic DNA synthesis
Source: Nucleic Acids Res. 2025 Feb 21;53(4):gkaf115. doi: 10.1093/nar/gkaf115 (PMC11840559; doi:10.1093/nar/gkaf115)
Supplement: gkaf115_Supplemental_File [file gkaf115_supplemental_file.docx]

**Supporting Information**

**Evolving a Terminal Deoxynucleotidyl Transferase for Commercial Enzymatic DNA Synthesis**

Stephanie M. Forget*, Mikayla J. Krawczyk, Anders M. Knight, Charlene Ching, Rachelle A. Copeland, Niusha Mahmoodi, Melissa Mayo, James Nguyen, Amanda Tan, Mathew Miller, Jonathan Vroom, Stefan Lutz*

Codexis Inc., 200 Penobscot Drive, Redwood City, CA 94063, USA

Corresponding authors: [stefan.lutz@codexis.com](mailto:stefan.lutz@codexis.com); [stephanie.forget@codexis.com](mailto:stephanie.forget@codexis.com)

**Supplementary Figure 1.** Initial activity with a 3’P-dNTP substrate. LC-MS analysis of reactions with various concentrations of purified TdT-01 with 2 μM TAATC, 100 μM 3’P-dATP, 37 °C, 16 h. Yields were calculated as the ion counts of TAATCA-3’P over unreacted TAATC.


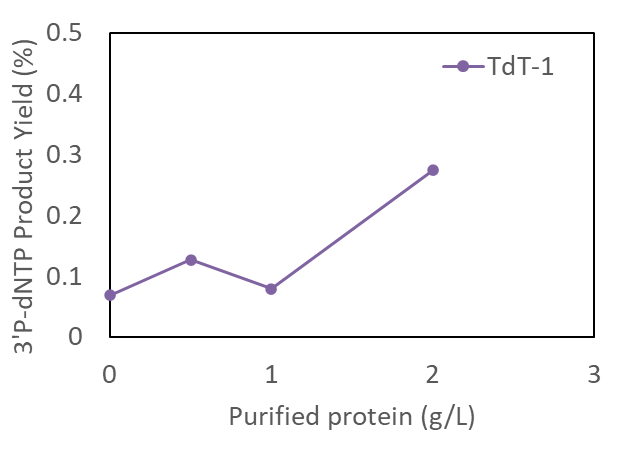


**Supplementary Figure 2.** Stability, solubility, and activity improvements in initial protein engineering rounds. Panels a and b show thermostability improvements in early protein engineering rounds. (a) Heat-treated HTP lysate (1h, 45-55C),reactions performed at with 25 vol% lysate, 37 °C, 1h with 2 mM TAATT and 100 mM ddTTP. (b) Heat-treated HTP lysates (1h, 48-56 °C), reactions performed with 25 vol% lysate, 49 °C, 1 h with 2 mM TAATT and 100 mM ddTTP. (c) SDS-PAGE of soluble and insoluble fraction of lyzed cells from early TdT variants. (d) Activity improvements over early protein engineering rounds with HTP lysates screened with 25 vol% lysate, 4 mM TTTTTTTATC and 200 mM 3’phos-dCTP reacted at 40 °C for 3h.


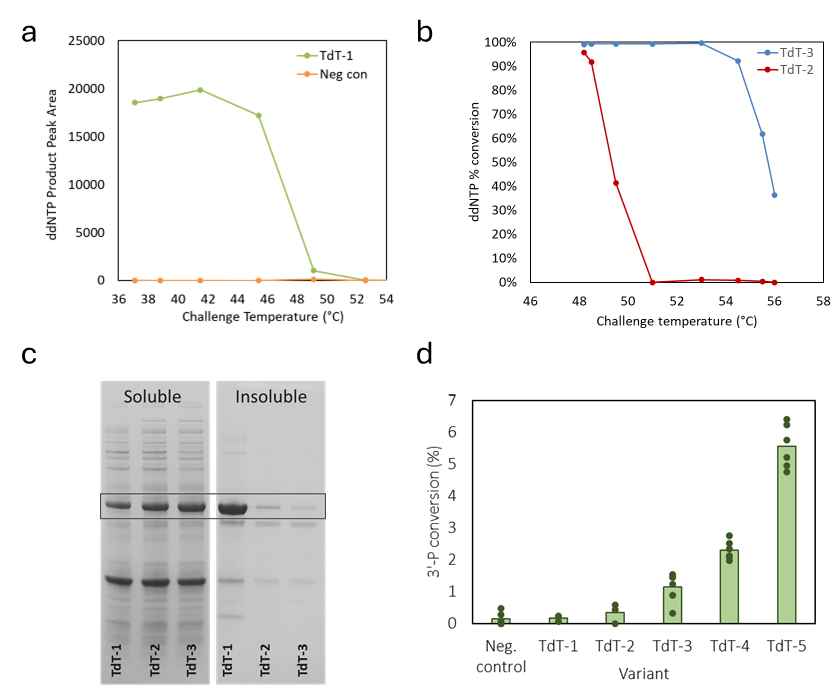


**Supplementary Figure** **3.** TdT activity following a thermostability pre-incubation challenge. Reactions contained 25 µM ddGTP, 1 µM oligo /56-FAM/TTTTTTTTTTTTTTTTTATC, 1 µM TdT variant (TdT-1, 7, 13, 18, 22, 26, or 32), 20 mM MOPS (pH 7.6), 50 mM KOAc, 0.25 mM CoCl_2_, 5% formamide, and 0.625% PEG-3350. Reactions were initiated by the addition of 5 µL oligo solution to 15 µL of all other heat-treated reaction components in a 96-well skirted PCR plate (Bio-Rad). Plates and solutions were kept cold prior and during initiation using aluminum blocks on ice. Reaction components containing all reaction components except oligonucleotide were heated for 15 min in a thermal cycler against a 41.8-72.4 °C horizontal temperature gradient. After the pre-incubation, the reaction plate was chilled, and the reaction was initiated by the addition of oligo solution. After vertexing to mix components and brief centrifugation, plates were reacted for 600s at 45 °C then heated at 95 °C for 2 minutes to inactivate the enzyme. Following heat-inactivation, reactions were further processed for analysis using the CE instrument.


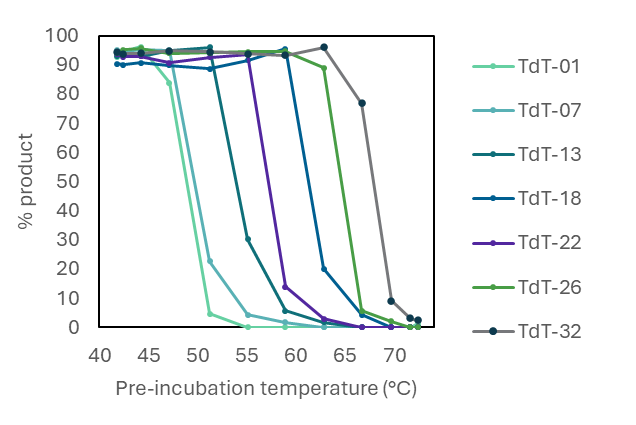


**Supplementary Table 1**. **Concentrations of TdT Proteins after Shake-flask Expression and Purification**

| **Source organism of TdT gene sequence** | **Soluble Enzyme Concentration After Purification [mg/mL]** |
| --- | --- |
| *Empidonax traillii*, truncated (**TdT-1**) | 8.0 |
| *Geospiza fortis*, truncated | 5.5 |
| *Serinus canaria*, truncated | 4.8 |
| *Ficedula albicollis*, truncated | 3.5 |
| *Mus musculus*, truncated | 2.4 |
| *Monodelphis domestica*, truncated | 2.3 |
| *Rattus norvegicus*, truncated | 1.5 |
| *Homo sapiens*, truncated | 1.5 |
| *Bos taurus*, truncated | 1.3 |
| *Monodelphis domestica* | 1.1 |
| *Homo sapiens* | 0.8 |
| *Bos taurus* | 0.8 |
| **TdT-32** | 29 |
| **TdT-33** | 4.3 (~15*) |

* Note: A PD-10 desalting column (Cytiva) was used for desalting for **TdT-33** in place of dialysis, which results in approximately 3.5-fold dilution of the purified protein compared to dialysis. The yield per mL was normalized here for comparison.

**Supplementary Table 2. Protein Purification Parameters**

| **Parameter** | **Volume** |
| --- | --- |
| Column volume | 5 mL |
| Flow rate | 5 mL/min |
| Pressure limit | 0.3 MPa |
| Sample volume | 35 mL |
| Equilibration volume | 5 column volumes (CV) = 25 mL |
| Wash unbound volume | 15 CV = 75 mL |
| Elution | Isocratic (step) |
| Elution volume | 5 CV = 25 mL |
| Fraction volume | 3 mL |
| Re‐equilibration volume | 5 CV = 25 mL |

**Supplementary Table 3. HPLC-MS/MS Analysis of Oligonucleotides – Method A (Agilent Ultivo)**

Quenched and clarified reaction supernatant (50 μL) was transferred to a 96-well PCR plate (Bio-Rad) for LC-MS analysis.  The samples were analyzed according to the Ultivo analytical method summarized in the table below.

| Instrument | | Agilent UHPLC 1260 prime series, Agilent Ultivo | | | | | |
| --- | --- | --- | --- | --- | --- | --- | --- |
| Column | | Phenomenex Clarity Oligo-MS 100A, 50 x 2.1 mm, 2.6 μm with Phenomenex Security Guard cartridge, C18, 2 mm ID | | | | | |
| Mobile Phases | | A: 50 mM TEAA = 50 mM triethylamine, 50 mM acetic acid, in water  B: acetonitrile  Needle wash: 80/20 water/acetonitrile | | | | | |
| Gradient | | Gradient from 98% A to 5% A and back again:   \| **Time (min)** \| **% A** \| \| --- \| --- \| \| 0.0 \| 98 \| \| 0.1 \| 98 \| \| 1.6 \| 5 \| \| 2.0 \| 5 \| \| 2.1 \| 98 \| \| 4.3 \| 98 \| | | | | | |
| Flow rate | | 0.6 mL/min | | | | | |
| Run time | | 4.3 min | | | | | |
| Peak retention times | | 1.58 min *(analytes co-elute)* | | | | | |
| Column temperature | | 50°C | | | | | |
| Injection volume | | 2 μL | | | | | |
| MS detection conditions | | Ion source: Agilent Jet Stream (AJS) ESI; scan type: SIM; polarity: negative; dwell time: 50 msec; fragmentor voltage: 180 V; time filter window: 0.02 | | | | | |
| MS source conditions | | Drying gas temperature: 325°C; drying gas flow: 7.0 L/min; nebulizer pressure: 35 psi; sheath gas temperature: 250 °C; sheath gas flow: 12.0 L/min; capillary voltage: -3500 V; nozzle voltage: -2000 | | | | | |
| Analyte charge state | | [M-3H]^3-^ | | | | | |
| **Product Name** | **Substrate Name** | | **dNTP** | **Method** | **Species** | **Analyte** | ***m/z*** |
| TAATTddT | TAATT | | ddTTP | 1 | substrate | TAATT | 737.1 |
|  |  | |  |  | product | TAATTddT | 881.2 |
| TAATCA-3'PO_4_ | TAATC | | 3'PO_4_-dATP | 2 | substrate | TAATC | 729.6 |
|  |  | |  |  | product | TAATCA-3'PO_4_ | 926.2 |
|  |  | |  |  | N+1 unblocked | TAATCA | 886.2 |
| TAATCT-3'NO_2_ | TAATC | | 3'NO_3_-dTTP | 3 | substrate | TAATC | 729.6 |
|  |  | |  |  | product | TAATCT-3'NO_2_ | 904.2 |
|  |  | |  |  | N+1 unblocked | TAATCT | 881.7 |
| TTTTTTTATCT-3'NO_2_ | TTTTTTTATC | | 3'NO_3_-dTTP | 4 | substrate | TTTTTTTATC | 989.8 |
|  |  | |  |  | product | TTTTTTTATCT-3'NO_2_ | 1106.2 |
|  |  | |  |  | N+1 unblocked | TTTTTTTATCT | 1091.2 |
| TTTTTTTATCC-3'PO_4_ | TTTTTTTATC | | 3'PO_4_-dCTP | 5 | substrate | TTTTTTTATC | 989.8 |
|  |  | |  |  | product | TTTTTTTATCC-3'PO_4_ | 1112.8 |
|  |  | |  |  | N+1 unblocked | TTTTTTTATCC | 1086.5 |
| TTTTTTTATCT-3'PO_4_ | TTTTTTTATC | | 3'PO_4_-dTTP | 6 | substrate | TTTTTTTATC | 990.1 |
|  |  | |  |  | product | TTTTTTTATCT-3'PO_4_ | 1118.1 |
|  |  | |  |  | N+1 unblocked | TTTTTTTATCT | 1091.2 |
| TTTTTTTATGC-3'PO_4_ | TTTTTTTATG | | 3'PO_4_-dCTP | 7 | substrate | TTTTTTTATG | 1003.2 |
|  |  | |  |  | product | TTTTTTTATGC-3'PO_4_ | 1126.2 |
|  |  | |  |  | N+1 unblocked | TTTTTTTATGC | 1099.5 |
| TTTTTTTATCG-3'PO_4_ | TTTTTTTATC | | 3'PO_4_-dGTP | 8 | substrate | TTTTTTTATC | 990.1 |
|  |  | |  |  | product | TTTTTTTATCG-3'PO_4_ | 1126.2 |
|  |  | |  |  | N+1 unblocked | TTTTTTTATCG | 1099.5 |
| TTTTTTTACAC-3'PO_4_ | TTTTTTTACA | | 3'PO_4_-dCTP | 9 | substrate | TTTTTTTACA | 993.3 |
|  |  | |  |  | product | TTTTTTTACAC-3'PO_4_ | 1115.8 |
|  |  | |  |  | N+1 unblocked | TTTTTTTACAC | 1089.2 |
| TTTTTTTATGT-3'PO_4_ | TTTTTTTATG | | 3'PO_4_-dTTP | 10 | substrate | TTTTTTTATG | 1003.7 |
|  |  | |  |  | product | TTTTTTTATGT-3'PO_4_ | 1131.7 |
|  |  | |  |  | N+1 unblocked | TTTTTTTATGT | 1105.1 |
| TTTTTTTACAG-3'PO_4_ | TTTTTTTACA | | 3'PO_4_-dGTP | 11 | substrate | TTTTTTTACA | 993.3 |
|  |  | |  |  | product | TTTTTTTACAG-3'PO4 | 1129.7 |
|  |  | |  |  | N+1 unblocked | TTTTTTTACAG | 1103.1 |
| TTTTTTTATGA-3'PO_4_ | TTTTTTTATG | | 3'PO_4_-dATP | 12 | substrate | TTTTTTTATG | 1003.7 |
|  |  | |  |  | product | TTTTTTTATGA-3'PO_4_ | 1134.7 |
|  |  | |  |  | N+1 unblocked | TTTTTTTATGA | 1108.1 |
| TTTTTTTCGGG-3'PO_4_ | TTTTTTTCGG | | 3'PO_4_-dGTP | 13 | substrate | TTTTTTTCGG | 1004 |
|  |  | |  |  | product | TTTTTTTCGGG-3'PO_4_ | 1140.4 |
|  |  | |  |  | N+1 unblocked | TTTTTTTCGGG | 1113.8 |
| TTTTTTTCTGC-3'PO_4_ | TTTTTTTCTG | | 3'PO_4_-dCTP | 14 | substrate | TTTTTTTCTG | 995.7 |
|  |  | |  |  | product | TTTTTTTCTGC-3'PO_4_ | 1118.7 |
|  |  | |  |  | N+1 unblocked | TTTTTTTCTGC | 1092.1 |
| TTTTTTTCGAC-3'PO_4_ | TTTTTTTCGA | | 3'PO_4_-dCTP | 15 | substrate | TTTTTTTCGA | 998.7 |
|  |  | |  |  | product | TTTTTTTCGAC-3'PO_4_ | 1121.7 |
|  |  | |  |  | N+1 unblocked | TTTTTTTCGAC | 1095.1 |
| TTTTTTTCCGC-3'PO_4_ | TTTTTTTCCG | | 3'PO_4_-dCTP | 16 | substrate | TTTTTTTCCG | 990.7 |
|  |  | |  |  | product | TTTTTTTCCGC-3'PO_4_ | 1113.7 |
|  |  | |  |  | N+1 unblocked | TTTTTTTCCGC | 1087.1 |
| TTTTTTTCGGC-3'PO_4_ | TTTTTTTCGG | | 3'PO_4_-dCTP | 17 | substrate | TTTTTTTCGG | 1004 |
|  |  | |  |  | product | TTTTTTTCGGC-3'PO_4_ | 1127 |
|  |  | |  |  | N+1 unblocked | TTTTTTTCGGC | 1100.4 |
| TTTTTTTACAA-3'PO_4_ | TTTTTTTACA | | 3'PO_4_-dATP | **18** | substrate | TTTTTTTACA | 993.3 |
|  |  | |  |  | product | TTTTTTTACAA-3'PO_4_ | 1124.4 |
|  |  | |  |  | N+1 unblocked | TTTTTTTACAA | 1097.8 |
|  |  | |  |  | N+2 unblocked | TTTTTTTACAAA | 1202.2 |
|  |  | |  |  | N+2 blocked | TTTTTTTACAAA-3'PO_4_ | 1228.8 |
| TTTTTTTATCC-3'PO_4_ | TTTTTTTATC | | 3'PO_4_-dCTP | **19** | substrate | TTTTTTTATC | 990.1 |
|  |  | |  |  | product | TTTTTTTATCC-3'PO_4_ | 1112.8 |
|  |  | |  |  | N+1 unblocked | TTTTTTTATCC | 1086.5 |
|  |  | |  |  | N+2 unblocked | TTTTTTTATCCC | 1182.5 |
|  |  | |  |  | N+2 blocked | TTTTTTTATCCC-3'PO_4_ | 1209.2 |
|  |  | |  |  | (N-1)+1 blocked | TTTTTTTATC-3'PO_4_ | 1017.0 |
| TTTTTTTACAA-3'PO_4_ | TTTTTTTACA | | 3'PO_4_-dATP | **20** | substrate | TTTTTTTACA | 993.3 |
|  |  | |  |  | product | TTTTTTTACAA-3'PO_4_ | 1124.4 |
|  |  | |  |  | N-1 unblocked | TTTTTTTAC | 888.9 |
|  |  | |  |  | N+1 unblocked | TTTTTTTACAA | 1097.8 |
|  |  | |  |  | N+2 blocked | TTTTTTTACAAA-3'PO_4_ | 1228.8 |
| TTTTTTTACAT-3'PO_4_ | TTTTTTTACA | | 3'PO_4_-dTTP | **21** | substrate | TTTTTTTACA | 993.3 |
|  |  | |  |  | product | TTTTTTTACAT-3'PO_4_ | 1121.4 |
|  |  | |  |  | N-1 unblocked | TTTTTTTAC | 888.9 |
|  |  | |  |  | N+1 unblocked | TTTTTTTACAA | 1097.8 |
|  |  | |  |  | N+2 blocked | TTTTTTTACAAT-3'PO_4_ | 1225.8 |
|  |  | |  |  | (N-1)+1 blocked | TTTTTTTACT-3'PO_4_ | 1017.0 |
| TTTTTTTATCA-3'PO_4_ | TTTTTTTATC | | 3'PO_4_-dATP | **22** | substrate | TTTTTTTATC | 990.1 |
|  |  | |  |  | product | TTTTTTTATCA-3'PO_4_ | 1121.0 |
|  |  | |  |  | N-1 unblocked | TTTTTTTAT | 894.0 |
|  |  | |  |  | N+1 unblocked | TTTTTTTATCC | 1086.5 |
|  |  | |  |  | N+2 blocked | TTTTTTTATCCA-3'PO_4_ | 1217.8 |
| TTTTTTTATCT-3'PO_4_ | TTTTTTTATC | | 3'PO_4_-dTTP | **23** | substrate | TTTTTTTATC | 990.1 |
|  |  | |  |  | product | TTTTTTTATCT-3'PO_4_ | 1118.1 |
|  |  | |  |  | N-1 unblocked | TTTTTTTAT | 894.0 |
|  |  | |  |  | N+1 unblocked | TTTTTTTATCC | 1086.5 |
|  |  | |  |  | N+2 blocked | TTTTTTTATCCT-3'PO_4_ | 1214.8 |
| TTTTTTTCGGG-3'PO_4_ | TTTTTTTCGG | | 3'PO_4_-dGTP | **24** | substrate | TTTTTTTCGG | 1004 |
|  |  | |  |  | product | TTTTTTTCGGG-3'PO_4_ | 1140.4 |
|  |  | |  |  | N+1 unblocked | TTTTTTTCGGG | 1113.8 |
|  |  | |  |  | N+2 unblocked | TTTTTTTCGGGG | 1140.4 |
|  |  | |  |  | N+2 blocked | TTTTTTTCGGGG-3'PO_4_ | 1250.1 |
|  |  | |  |  | (N-1)+1 blocked | TTTTTTTCGG-3'PO_4_ | 1030.6 |

**Supplementary Table 4. HPLC-MS/MS Analysis of Oligonucleotides – Method B (LTQ)**

Quenched and clarified reaction supernatant (50 μL) was then transferred to a 96-well skirted PCR plate for LC-MS analysis.  The samples were analyzed according to the LTQ analytical method summarized in the table below.

| Instrument | | Thermo Accela, Thermo LTQ XL | | | | | |
| --- | --- | --- | --- | --- | --- | --- | --- |
| Column | | Phenomenex Clarity Oligo-MS 100A, 50 x 2.1 mm, 2.6 μm with Phenomenex Security Guard cartridge, C18, 2 mm ID | | | | | |
| Mobile Phases | | A: 389 mM hexafluoroisopropanol (HFIP), 13.6 mM triethylamine (TEA), in water  B: mobile phase A diluted 50/50 in methanol (194 mM HFIP, 6.8 mM TEA, in 50% methanol)  Needle wash: 95/5 water/acetonitrile | | | | | |
| Gradient | | Gradient from 98% A to 5% A and back again:   \| **Time (min)** \| **% A** \| \| --- \| --- \| \| 0.0 \| 98 \| \| 0.5 \| 98 \| \| 1.0 \| 5 \| \| 2.0 \| 5 \| \| 2.25 \| 98 \| \| 4.5 \| 98 \| | | | | | |
| Flow rate | | 0.4 mL/min | | | | | |
| Run time | | 4.5 min | | | | | |
| Peak retention times | | 2.0 min (analytes co-elute) | | | | | |
| Column temperature | | 35 °C | | | | | |
| Injection volume | | 10 μL | | | | | |
| MS detection conditions | | Scan type: SIM; polarity: negative; scan rate: normal | | | | | |
| MS source conditions | | Sheath gas: 20; aux gas: 10; sweep gas: 0; spray voltage: 5; capillary temperature: 375 °C; capillary voltage: -6 | | | | | |
| Analyte charge state | | [M-2H]^2-^ | | | | | |
| **Product Name** | **Substrate Name** | | **dNTP** | **Method** | **Species** | **Analyte** | ***m/z*** |
| TTTTTTTATCT-3'PO_4_ | TTTTTTTATC | | 3'PO_4_-dTTP | LTQ 1 | substrate | TTTTTTTATC | 1485.3 |
|  |  | |  |  | product | TTTTTTTATCT-3'PO_4_ | 1677.2 |
| TTTTTTTATCG-3'PO_4_ | TTTTTTTATC | | 3'PO_4_-dGTP | LTQ 2 | substrate | TTTTTTTATC | 1485.5 |
|  |  | |  |  | product | TTTTTTTATCG-3'PO_4_ | 1689.5 |
| TTTTTTTACAC-3'PO_4_ | TTTTTTTACA | | 3'PO_4_-dCTP | LTQ 3 | substrate | TTTTTTTACA | 1489.8 |
|  |  | |  |  | product | TTTTTTTACAC-3'PO_4_ | 1674.3 |

**Supplementary Table 5. RapidFire SPE-MS Conditions for TAATC Detection**

| **Agilent RapidFire Conditions** | | | | | | |
| --- | --- | --- | --- | --- | --- | --- |
| Buffer A | 15% 25mM ammonium acetate pH 5.7 LC/MS grade water, 85% acetonitrile LC/MS grade; 0.8mL/min flow rate (Pump1) | | | | | |
| Buffer B | 40% methanol LC/MS grade, 60% 10mM ammonium bicarb pH 8 LC/MS grade; 1.25mL/min flow rate (Pump2) | | | | | |
| Buffer C | 40% methanol LC/MS grade, 60% 10mM ammonium bicarb pH 8 LC/MS grade; 0.8mL/min flow rate (Pump3) | | | | | |
| Aqueous wash | Water | | | | | |
| Organic wash | Acetonitrile | | | | | |
| SPE cartridge | Agilent RapidFire cartridge H (HILIC) | | | | | |
| RF state 1 | 600 ms | | | | | |
| RF state 2 | 4000 ms | | | | | |
| RF state 3 | 1000 ms | | | | | |
| RF state 4 | 3000 ms | | | | | |
| RF state 5 | 4000 ms | | | | | |
| **Agilent Jet Stream Source Parameters** | | | | | | |
| Drying gas temperature | 300 °C | | | | | |
| Drying gas flow | 10 L/min | | | | | |
| Nebulizer pressure | 40 psi | | | | | |
| Sheath gas temperature | 250 °C | | | | | |
| Sheath gas flow | 10L/min | | | | | |
| Capillary voltage | -2500 V | | | | | |
| Nozzle voltage | -2000 V | | | | | |
| **Agilent 6470 Triple Quadrupole MRM Parameters** | | | | | | |
| **Compound** | **Q1** | **Q3** | **Dwell** | **Fragmentor** | **CE** | **CAV** |
| TAATC_729.8 | 729.8 | 729.8 | 82 | 160 | 0 | 4 |
| TAATC_729.8 | 729.8 | 714.2 | 82 | 160 | 24 | 4 |
| TAATC_729.8 | 729.8 | 610.3 | 82 | 160 | 26 | 4 |

**Supplementary Table 6. RapidFire - Detailed HILIC SPE Conditions**

| **SPE Condition** | **Detailed SPE Conditions** |
| --- | --- |
| **1** | 90% ACN 10% 25mM Ammonium Acetate pH5.8_load, 50%ACN 10% Ammonium bicarb pH 7.5 elute |
| **2** | 90% ACN 10% 25mM Ammonium Acetate pH5.8_load, 50%ACN 10% Ammonium bicarb pH 8 elute |
| **3** | 85% ACN 15% 25mM Ammonium Acetate pH5.8_load, 50%ACN 10% Ammonium bicarb pH 7.5 elute |
| **4** | 85% ACN 15% 25mM Ammonium Acetate pH5.8_load, 60%ACN 10% Ammonium bicarb pH 7.5 elute |
| **5** | 85% ACN 15% 25mM Ammonium Acetate pH5.8_load, 40%ACN 10% Ammonium bicarb pH 7.5 elute |
| **6** | 0.6mL-min 85% ACN 15% 25mM Ammonium Acetate pH5.8 load; 1S Extra wash; 60%ACN 10% Ammonium bicarb pH 7.5 elute |
| **7** | 0.6mL-min 85% ACN 15% 25mM Ammonium Acetate pH5.8 load; NO Extra wash; 60%ACN 10% Ammonium bicarb pH 7.5 elute |
| **8** | 0.4mL-min 85% ACN 15% 25mM Ammonium Acetate pH5.8 load; NO Extra wash; 60%ACN 10% Ammonium bicarb pH 7.5 elute |
| **9** | 0.4mL-min 85% ACN 15% 25mM Ammonium Acetate pH5.8 load; 1S Extra wash; 60%ACN 10% Ammonium bicarb pH 7.5 elute |
| **10** | 0.6mL-min 80% ACN 20% 25mM Ammonium Acetate pH5.8 load; 1S Extra wash; 60%ACN 10% Ammonium bicarb pH 7.5 elute |

**Supplementary Table 7. RapidFire – Final SPE-MS Conditions for TTTTTTTATC Detection**

| **Agilent RapidFire Conditions** | | | | | |  |
| --- | --- | --- | --- | --- | --- | --- |
| Buffer A | 20% 20mM ammonium acetate pH 5.8 LC/MS grade water, 80% acetonitrile LC/MS grade; 0.6mL/min flow rate (Pump1) | | | | |  |
| Buffer B | 70% acetonitrile LC/MS grade, 20% water LC/MS grade, 10% 10mM ammonium bicarb pH 8.6 LC/MS grade; 1.25mL/min flow rate (Pump2) | | | | |  |
| Buffer C | 70% acetonitrile LC/MS grade, 20% water LC/MS grade, 10% 10mM ammonium bicarb pH 8.6 LC/MS grade; 0.8mL/min flow rate (Pump3) | | | | |  |
| Aqueous wash | Water | | | | |  |
| Organic wash | Acetonitrile | | | | |  |
| SPE cartridge | Agilent RapidFire cartridge H6 (HILIC) | | | | |  |
| RF state 1 | 600 ms | | | | |  |
| RF state 2 | 4000 ms | | | | |  |
| RF state 3 | 1000 ms | | | | |  |
| RF state 4 | 3000 ms | | | | |  |
| RF state 5 | 4000 ms | | | | |  |
| **Agilent Jet Stream Source Parameters** | | | | | |  |
| Drying gas temperature | 325 °C | | | | |  |
| Drying gas flow | 10 L/min | | | | |  |
| Nebulizer pressure | 20 psi | | | | |  |
| Sheath gas temperature | 275 °C | | | | |  |
| Sheath gas flow | 10 L/min | | | | |  |
| Capillary voltage | -2500 V | | | | |  |
| Nozzle voltage | -2000 V | | | | |  |
| **Agilent 6470 Triple Quadrupole SRM parameters** | | | | | |  |
| **Compound** | **Q1** | **Dwell** | **Fragmentor** | **CAV** | **Polarity** | |
| TTTTTTTATC_990.3 | 990.3 | 150 | 160 | 3 | Neg | |
| TTTTTTTATC_742.5 | 742.5 | 150 | 130 | 4 | Neg | |

**Supplementary Table 8. RapidFire - SPE-MS/MS Conditions for TTTTTTTATC-G3’P Detection**

| **Agilent RapidFire Conditions** | | | | | |
| --- | --- | --- | --- | --- | --- |
| Buffer A | A mixture of 75% LC/MS grade acetonitrile and 25% of 50mM ammonium acetate pH 5.8; 1.5 mL/min flow rate | | | | |
| Buffer B | A mixture of 60% LC/MS grade acetonitrile, 30% of LC/MS grade water, and 10% of 50mM LC/MS grade ammonium bicarbonate pH 8.0; 1.5 mL/min flow rate | | | | |
| Buffer C | A mixture of 70% LC/MS grade acetonitrile, 30% of 50mM LC/MS grade ammonium bicarbonate pH 8.0; 0.8 mL/min flow rate | | | | |
| Aqueous wash | Water | | | | |
| Organic wash | Acetonitrile | | | | |
| SPE cartridge | Agilent RapidFire cartridge H6 | | | | |
| RF state 1 | 120 ms | | | | |
| RF state 2 | 5000 ms | | | | |
| RF state 3 | 1500 ms | | | | |
| RF state 4 | 4500 ms | | | | |
| RF state 5 | 4000 ms | | | | |
| **Agilent Jet Stream Source Parameters** | | | | | |
| Drying gas temperature | 325 °C | | | | |
| Drying gas flow | 10 L/min | | | | |
| Nebulizer pressure | 20 psi | | | | |
| Sheath gas temperature | 325 °C | | | | |
| Sheath gas flow | 10 L/min | | | | |
| Capillary voltage | -5000 V | | | | |
| Nozzle voltage | -2000 V | | | | |
| **Agilent 6470 Triple Quadrupole MS2 SIM Parameters** | | | | | |
| **RF Method** | **Product** | ***m/z* (3-) (Quantifier)** | **RT** | ***m/z* (4-)** | **RT** |
| 1 | TTTTTTTATCG-3'PO_4_ | 1126.7 | 0.151 | 844.8 | 0.151 |
| 2 | TTTTTTTATCT-3'PO_4_ | 1118.2 | 0.151 | 834.5 | 0.163 |

| 3 | TTTTTTTATCC-3'PO_4_ | 1113.2 | 0.151 | 834.5 | 0.163 |
| --- | --- | --- | --- | --- | --- |
| 4 | TTTTTTTACAT-3'PO_4_ | 1121.4 | 0.185 | 840.8 | 0.188 |
| 5 | TTTTTTTATGA-3'PO_4_ | 1134.7 | 0.164 | 850.8 | 0.157 |
| 6 | TTTTTTTACAG-3'PO_4_ | 1129.7 | 0.160 | 847.1 | 0.164 |
| 7 | TTTTTTTCGGG-3'PO_4_ | 1127.1 | 0.143 | 845.1 | 0.145 |

Signal of TTTTTTTATC significantly increased when loading buffer composition changed from 10% of 25mM ammonium acetate, pH 5.8 (condition 2) to 15% of 25 mM ammonium acetate, pH 5.8 (condition 3). Similar sequences of oligonucleotides can be retained and detected with 10%-30% of aqueous ammonium acetate buffer at 50mM between pH 5.5 – 5.8.

| **Supplementary Table 9. TdT variant amino-acid sequences (FASTA format)**  >TdT-1  MHHHHHHGGSGHPPSNTPELEVPSVIARKVSQYSCQRKTTLNNYNKKFTDAFEIMAENYEFKENEIFCLEFLRAASLLKYLPFPVTRMKDIQGLPCIGDQVRDVIEEIIEEGESSRVKEVLNDERYKAFKQFTSVFGVGVKTSEKWFRMGLRTVEELKADKSLKLSKMQKAGFLYYEDLVSCVSKAEADAVTLIVKNTVSTFLPDALVTITGGFRRGKKMGHDIDFLITNPGPKEDDELLHKVVDLLKKQGLLLYCDIIESTFVEEQLPSRKIDAMDNFQKCFAILKLYQPGVDNSSYNTSKKSDMAEVKDWKAIRVDLVITPFEQYAYALLGWTGSREFGRDLRRYASHERKMILDNHGLYDRKKRIFLKAGSEEEIFAHLGLDYVEPWERNA |
| --- |
| >TdT-2  MHHHHHHGGSGHPPSNTPEGGVPSVIARKVSQYSCQRKTTLNNYNKKFTDAFEIMAENYEFKENEIFRLEFLRAASLLKYLPFPVTRMKDIQGLPCIGDQVRDVIEEIIEEGESSRVKEVLNDERYKAFKQFTSVFGVGVKTSEKWFRMGLRTVEELKASKSLKLSKMQKAGFLYYEDLASCVSKAEADAVTLIVKNTVRTFLPDALVTITGGFRRGKKMGHDIDFLITNPGPKEDDELLHKVVDGLKKQGLLLYCDIIESTFVEEQLPSRKIDAMDNFQKCFAILKLYQPGVDNSSYNTSKKSDMAEVKDWKAIRVDLVITPFEQYAYALLGWTGSREFGRDLRRYASHERKMILDNHGLYDRKKRIFLKAGSEEEIFAHLGLDYVEPWERNA |
| >TdT-3  MHHHHHHGGSGHPPSNTPEGGVPSVIARKVSQYSCQRKTTLNNYNKKFTDAFEIMAENYEFKENEIARLEFLRAASLLKYLPFPVTSMKDIQGLPCIGDQVRDVIEEIIETGESSRVKEVLNDERYKAFKQFTSVFGVGVKTSEKWFRMGLRTVEEAKASKSLKLSKMQKAGFLYYEDLASCVSKAEADAVTLIVKNTVRTFLPDALVTITGGFRRGKKMGHDIDFLITNPGPKEDDELLHKVVDGLKKQGLLLYCDIIESTFVEEQLPSRKIDAMDNFQKCFAILKLYQPGVDNSSYNTSKKSDMAEVKDWKAVRVDLVITPFEQYAYALLGWTGSREFGRDLRRYASHERKMILDNHGLYDRKKRIFLKAGSEEEIFAHLGLDYVEPWERNA |
| >TdT-4  MHHHHHHGGSGHPPSNTPEGGVPSVIARKVSQYSCQRKTTLNNYNKKFTDAFEIMAENYKFKENEIFCLEFKRAASLLKYLPFPVTRMKDIQGLPCIGDQVRDVIEEIIEEGESSRVKEVLNDERYKAFKQFTSVFGVGVKTSEKWFRMGLRTVEELKASKSLKLSKMQKAGFLYYEDLASCVSKAEADAVTLIVKNTVRTFLPDALVTITGGFRRGKKMGHDIDFLITNPGPKEDDELLHKVVDGLKKQGLLLYCDIKESTFVEEQLPSRKIDAMDNFQKCFAILKLYQPGVDNSSYNTSKKSDMAEVKDWKAIRVDLVITPFEQYAYALLGWTGSKEFGRDLRRYASHERKMILDRHGLYDRKKRIFLKAGSEEEIFAHLGLDYVEPWERNA |
| >TdT-5  MHHHHHHGGSGHPPSNTPEGEVPSVIARKVSQYSCQRKTTLNNYNKKFTDAFEIMAENYKFKENEIACLEFKRAASLLKYLPFPVTSMKDIQGLPCIGDQVRDVIEEIIEEGESSRVKEVLNDERYKAFKQFTSVFGVGVKTSEKWFRMGLRTVEEAKASKSLKLSKMQKAGFLYYEDLASCVSKAEADAVTLIVKNTVRTFLPDALVTITGGFRRGKKMGHDIDFLITNPGPKEDDELLHKVVDLLKKQGLLLYCDIKESTFVEEQLPSRKIDAMDNFQKCFAILKLYQPGVDNSSYNTSKKSDMAEVKDWKAIRVDLVITPFEQYAYALLGWTGSKEFGRDLRRYASHERKMILDRHGLYDRKKRIFLKAGSEEEIFAHLGLDYVEPWERNA |
| >TdT-6  MHHHHHHGGSGHPPSNTPEGEVPSVIARKVSQYSCQRKTTLNNYNKKFTDAFEIMAENYKFKENEIACLEFKRAASLLKYLPFPVTRMKDIQGLPCIGDQVRDVIEEIIEEGESSRVKEVLNDERYKAFKQFTSVFGVGVKTSEKWFRMGLRTVEELKASKSLKLSKMQKAGFLYYEDLVSCVSKAEADAVTLIVKNTVRTFLPDALVTITGGFRRGKKMGHDIDFLITNPGPKEDDELLHKVVDGLKKQGLLLYCDIKESTFVEEQLPSRKIDAMDNFQKCFAILKLYQPGVDNSSYNTSKKSDMAEVKDWKAVRVDLVITPFEQYAYALLGWTGSKEFGRDLRRYASHERKMILDRHGLYDRKKRIFLKAGSEEEIFAHLGLDYVEPWERNA |
| >TdT-7  MHHHHHHGGSGHPPSNTPEGEVPSVIARKVSQYSCQRKTTLNNYNKKFTDALEIMAENYKFKENEIACLEFKRAASLLKYLPFPVTSMKDIQGLPCIGDQVRDVIEEIIEEGESSRVKEVLNDERYKAFKQFTSVFGVGVKTSEKWFRMGLRTVEEAKASKSLKLSKMQKAGFLYYEDLASCVSKAEADAVTLIVKNTVRTFLPDALVTITGGFRRGKKMGHDIDFLITNPGPKEDDELLHKVVDLLKKQGLLLYCDIKESTFVEEQLPSRKIDAMDNFQKCFAILKLYQPGVDNSSYNTSKKSDMAEVKDWKAIRVDLVITPFEQYAYALLGWTGSKEFGRDLRRYAEEERKMILDRHGLYDRKKRIFLKAGSEEEIFAHLGLDYVEPWERNA |
| >TdT-8  MHHHHHHGGSGHPPSNTPEGEVPSVIARKVSQYSCQRKTTLNNYNKKFTDALEIMAENYKFKENEIACLEFKRAASLLKYLPFPVTSMKDIQGLPCIGDQVRDVIEEIIEEGESSRVKEVLNDERYKAFKQFTSVFGVGVKTSEKWFRMGLRTVEEAKASKSLKLSKMQKAGFLYYEDLASCVSKAEADAVTLIVKNTVRTFLPDALVTITGGFRRGKPMGHDIDFLITNPGPKEDDELLHKVVDLLKKQGLLLYCDIKESTFVEEQLPSRKIDAMDNFQKCFAILKLYQPGVDNSSYNTSKKSDMAEVKDWKAIRVDLVITPFEQYAYALLGWTGSKEFGRDLRRYAEEERKMILDNHGLYDRKKRIFLKAGSEEEIFAHLGLDYVEPWERNA |
| >TdT-9  MHHHHHHGGSGHPPSNTPEGEVPSVIARKVSQYSCQRKTTLNNYNKKFTDALEIMAENYKFKENLIACLEFKRAASLLKYLPFPVTSMKDIQGLPCIGDQVRDVIEEIIEEGESSRVKEVLNDERYKAFKQFTSVFGVGVKTSEKWFRMGLRTVEEAKASKSLKLSKMQKAGFLYYEDLASCVSKAEADAVTLIVKNTVRTFLPDALVTITGGFRRGKPMGHDIDFLITNPGPKEDDELLHKVVDLLKKQGLLLYCDIKESTFVEEQLPSRKIDAMDNFQKCFAILKLYQPGVDNSSYNTSKKSDMAEVKDWKAIRVDLVITPFEQYAYALLGWTGSKEFGRDLRRYAEEERKMILDNHGLYDRKKRIFLKAGSEEEIFAHLGLDYVEPWERNA |
| >TdT-10  MHHHHHHGGSGHPPSNTPEGEVPSVIARKVSQYSCQRKTTLNNYNKKFTDALEIMAENYKFKENLIACLEAKRAASILKYLPFPVTSMKDIQGLPCIGDQVRDVIEEIIEEGESSRVKEVLNDERYKAFKQFTSVFGVGVKTSEKWFRMGLRTVEEAKASKSLKLSKMQKAGFLYYEDLASCVSKAEADAVTLIVKNTVRTFLPDALVTITGGFRRGKPMGHDIDFLITNPGPKEDDELLHKVVDLLKKQGLLLYCDIKESTFVEEQLPSRKIDAMDNFQKCFAILKLYQPGVDNSSYNTSKKSDMAEVKDWKAIRVDLVITPFEQYAYALLGWTGSKEFGRDLRRYAEEERGMILDNHGLYDRKKRIFLKAGSEEEIFAHLGLDYVEPWERNA |
| >TdT-11  MHHHHHHGGSGHPPSNTPEGEVPSVIARKVSQYSCQRKTTLNNYNKKFTDALEIMAENYKFKENLIACLEAKRAASILKYLPFPVTSMKDIQGLPCIGDQVRDVIEEIIEEGESSRVKEVLNDERYKAFKQFTSVFGVGVKTSEKWFRMGLRTVEEAKASKSLKLSKMQKAGFLYYEDLASRVSKEEADAVTLIVKNTVRTFLPDALVTITGGFRRGKPMGHDIDFLITNPGPKEDDELLHKVVDLLKKQGLLLYEDIKESTFVEEQLPSRKIDAMDNFQKCFAILKLYQPGVDNSSYNTSKKSDMAEVKDWKAIRVDLVITPFEQYAYALLGWTGSKEFGRDLRRYAEEERGMILDNHALYDRKKRIFLKAGSEEEIFAHLGLDYVEPWERNA |
| >TdT-12  MHHHHHHGGSGHPPSNTPEGEVPSVIARKVSQYSCQRKTTLNNYNKKFTDALEIMAENYKFKENLIACLEAKRAASILKYLPFPVTSMKDIQGLPCIGDQVRDVIEEIIEEGESSRVKEVLNDERYKAFKQFTSVFGVGVKTSEKWFRMGLRTVEEAKASKSLKLSKMQKAGFLYYEDLASRVSKEEADAVTLIVKNTVRTFLPDALVTITGGFRRGKPMGHDIDFLITNPGPKEDDELLHKVVDLLKKQGLLLYEDIKESTFVEEQLPSRKIDAMDNFQKCFAILKLYQPGVDNSSYNTSKKSDMAEVKDWKAIRVDLVITPFEQYAYALLGWTGSKEFRRDLRRYAEEERGMILDNHALYDRKKRIFLKAGSEEEIFAHLGLDYVEPWERNA |
| >TdT-13  MHHHHHHGGSGHPPSNTPEGEVPSVIARPVSQYSCQRKTTLNNYNKKFTDALEIMAENYKFKENLIACLEAKRAASILKYLPFPVTSMKDIQGLPCITDQVRDVIEEIIEEGESSRVKEVLNDERYKAFKQFTSVFGVGVKTAEKWFRMGLRTVEEAKASKSLKLSKMQKAGFLYYEDLASRVSKEEADAVTLIVKNTVRTFLPDALVTITGGFRRGKPMGHDIDFLITNPGPKEDDELLHKVVDLLKKQGLLLYEDIKESTFVEGQLPSRKIDAMDNFQKCFAILKLYQPGVDNSSYNTSKKSDMAEVKDWKAIRVDLVITPFEQYAYALLGWTGSKEFRRDLRRYAEEERGMILDNHALYDRKKRIFLKAGSEEEIFAHLGLDYVEPWERNA |
| >TdT-14  MHHHHHHGDSGHPPSNTPEGEVPSVIARPVSQYSCQRKTTLNNYNKKFTDALEIMAENYKFKENLIACLEAKRAASILKYLPFPVTSMKDIQGLPCITDQVRDVIEEIIEEGESSRVKEVLNDERYKAFKQFTSVFGVGVKTAEKWFRMGLRTVEEAKASKSLKLSKMQKAGFLYYEDLASRVSKEEADAVTLIVKNTVRTFLPDALVTITGGFRRGKPMGHDIDFLITNPGPKEDDELLHKVVDLLKKQGLLLYEDIKESTFVEGQLPSRKIDAMDNFQKCFAILKLYQPGVDNSSYNTSKKSDMAEVKDWKAIRVDLVITPFEQYAYALLGWTGSKEFRRDLRRYAEEERGMILDNHALYDLKKRIFLKAGSEEEIFAHLGLDYVEPWERNA |
| >TdT-15  MHHHHHHGDSGHPPSNTPEGEVPSVIARPVSQYSCQRKTTLNNYNKKFTDALEIMAENYKFKENLIACLEAKRAASILKYLPFEVTSMKDIQGLPCITDQVRDVIEEIIEEGESSRVKEVLNDERYKAFKQFTSVFGVGVKTAEKWFRMGLRTVEEAKASKSLKLSKMQKAGFLYYEDLASRVSKEEADAVTLIVKNTVRTFLPDALVTITGGFRRGKPMGHDVDFLITNPGPKEDDELLHKVVDLLKKQGLLLYEDIKESTFVEGQLPSRKIDAMDNFQKCFAILKLYQPGVDNSAVGTSKKSDMAEVKDWKAIRVDLVITPFEQYAYALLGWTGSKEFRRDLRRYAEEERGMILDNHALYDLKKRIFLKAGSEEEIFAHLGLDYVEPWERNA |
| >TdT-16  MHHHHHHGGSGHPPSNTPEGEVPSVIARPVSQYSCQRKTTLNNYNKKFTDALEIMAENYKFKENLIACLEAKRAASILKYLPFEVTSMKDIQGLPCITDQVRDVIEEIIEEGESSRVKEVLNDERYKAFKQFTSVFGVGVKTAEKWFRMGLRTVEEAKASKSLKLSKMQKAGFLYYEDLASRVSKEEADAVTLIVKNTVRTFLPDALVTITGGFRRGKPMGHDVDFLITNPGPKEDDELLHKVVDLLKKQGLLLYEDIKESTFVEGQLPSRKIDAMDNFQKCFAILKLYQPGVDNSAVGTSKKSDMAEVKDWKAIRVDLVITPFEQYAYALLGWTGSKEFRRDLRRYAEEERGMILDNHALYDLKKRIFLKAGSEEEIFAHLGLDYVEPWERNA |
| >TdT-17  MHHHHHHGGSGHPPSNTPEGEVPSVIARPVSQYACQRKTTLNNYNKKFTDALEIMAENYKFKENLIACLEAKRAASIFKYLPFEVTSMKDIQGLPCITDQVRDVIEEIIEEGESSRVKEVLNDERYKAFKQFTSVFGVGVKTAEKWYRMGLRTVEEAKASKSLKLSKMQKAGFLYYEDLASMVSKEEADAVTLIVKNTVRTFLPDALVTITGGFRRGKPMGHDVDFLITNPGRKEDDELLHKVVDLLKKQGLLLYEDIKESTFVEGQLPSHKIDAMDNFQKCFAILKLYQPGVDNSAVGTSKKSDMAEVKDWKAIRVDLVITPFEQYAYALLGWTGSKQFRRDLRRYAEEERGMILDNHALYDLKKRIFLKAGSEEEIFAHLGLDYVEPWERNA |
| >TdT-18  MHHHHHHGGSGHPPSNTPEKEVPSVIARPVSQYACQRKTTLNNYNKKFTDALEIMAENYKFKENLIACLEAKRAASIFKYLPFEVTSMKDIQGLPCITDQVRDVIEEIIEEGESSRVKEILNDERYKAFKQFTSVFGVGVKTAEKWYRMGLRTVEEAKASKSLKLSKMQKAGFLYYEDLASMVSKEEADAVTLIVKETVRTFLPDALVTITGGFRRGKPMGHDVDFLITNPGRKEDDELLHKVVDLLKKQGLLLYEDIKESTFVEGQLPSHKIDAMDNFQKCFAILKLYQPKVDNSAVGTSKKSDMAEVKDWKAIRVDLVITPFEQYAYALLGWTGSKQFRRDLRRYAEEERGMILDNHALYDLKKRIFLKAGSEEEIFAHLGLDYVEPWERNA |
| >TdT-19  MHHHHHHGGSGHPPSNTPEKEVPSVIARPVSQYACQRKTTLNNYNKKFTDALEIMAENYKFKENLIACLEAKRAASIFKSLPFEVTSMKDIQGLPCITDQVRDVIEEIIEEGESSRVKEILNDERYKAFKQFTSVFGVGVKTSEKWYRMGLRTVEEAKASKSLKLSKMQKAGFLYYEDLASMVSKEEADAVTLIVKETVRTFVPDALVTIAGGFRRGKPMGHDVDFLITNPGRKEDDELLHKVVDLLKKQGLLLYEDIKESTFVEGQLPSHKIDAMDNFQKCFAILKLYQPKVDNSAVGTSKKSDMAEVKDWKAIRVDLVITPFEQYAYALLGWTGSGQFRRDLRRYAEEERGMILDNHALYDLKKRIFLKAGSEEEIFAHLGLDYVEPWERNA |
| >TdT-20  MHHHHHHGGSGHPPSNTPEKEVPSVIARPVSQYACQRKTTLNNYNKKFTDALEIMAENYKFKENLQACLEAKRAASIFKSLPFEVTSMKDIQGLPCITDDVRDVIEEIIEEGESSRVKEILNDERYKAFKQFTSVFGVGVKTSEKWYRMGLRTVEEAKASKSLKLSKMQKAGFLYYEDLASMVSKEEADAVTLIVKETVRTFVPDALVTIAGGFRRGKPMGHDVDFLITNPGRKMDDELLHKVVDLLKKQGLLLYEDIKESTFVEGQLPSHKIDAMDNFQKCFAILKLYQPKVDNSAVGTSKKSDMAEVKDWKAVRVDLVITPFEQFAYALLGWTGSGQFRRDLRRYAEEERDMLLDNHALYDLKKRIFLKAGSEEEIFAHLGLDYVEPWERNA |
| >TdT-21  MHHHHHHGGSGHPPSNTPEKEVPSVIARPVSQYACQRKTTLNNYNKKFTDALEIMAENYKFKENLQACLEAKRAASIFKSLPFEVTSMKDIQGLPCITDDVRDVIEEIIEEGESSRVKEILNDERYKAFKQFTSVFGVGVKTSEKWYRMGLRTVEEAKASKSLKLSKMQKAGFLYYEDLASMVSKEEADAVTLIVKETVRTVVPDALVTIAGGFRRGKPMGHDVDFLITNPGRKEDDELLHKVVDLLKKQGLLLYEDIKESTFVEGQLPSHKIDAMDNFQKCFAILKLYQPKVDNSAVGTSKKSDMAEVKDWKAVRVDLVITPFEQFAYALLGWTGSGQFRRDLRRYAEEERDMLLDNHRLYDLKKGIFLSAGSEEEIFAHLGLDYVEPWERNA |
| >TdT-22  MHHHHHHGGSGHPPSNTPEKEVPSVIARPVSQYACQRKTTLNNYNKKFTDALEIMAENYKFKENLQACLEAKRAASIFKSLPFEVTSMKDIQGLPCITDDVRDVIEEIIEEGESSRVKEILNDERYKAFKQFTSVFGVGVKTSEKWYRMGLRTVEEAKASKSLKLSKMQKAGFLYYEDLASMVSKEEADAVTLIVKETVRTVVPDALVTIAGGFRRGKPMGHDVDFLITNPGRKEDDELLHKVVDLLKKQGLLLYEDIRESTFVEGQLPSHKIDALDNFQKCFAILKLYQPKVDNSAVGTSKKSDMAEVKDWKAVRVDLVITPFEQFAYALLGWTGSGQFRRDLRRYAEEERDMLLDNHRLYDLKKGIFLSAGSEEEIFAHLGLDYLEPWERNA |
| >TdT-23  MHHHHHHGGSGHPPSNTPEKEVPSVIARHVSQYACQRKTTLNNYNKKFTDALEIMAENYKFKENLQACLEAKRAASIFKSLPFEVTSMKDIQGLPCITDDVRDVIEEIIEEGESSRVKEILNDERYKAFKQFTSVFGVGVKTSEKWYRMGLRTVEEAKASKSLKLSKMQKAGFLYYEDLASGVSKEEADAVTLIVKETVRTVVPDALVTIAGGFRRGKPMGHDVDFLITNPGRPEDDELLHKVVDLLKKQGLLLYEDIRESTFVEGQLPSPKIDALDNFQKCFAILKLYQPKVDNSAVGTSKKSDMAEVKDWKAVRVDLVITPFEQFAYALLGWTGSGQFRRDLRRYAEEERDMLLDNHRLYDLKKGIFLSAGSEEEIFGHLGLDYLEPWERNA |
| >TdT-24  MHHHHHHGGSGHPPSNTPEKEVPSVIARHVSQYACQRKTTLNNYNKKFTDALEIMAENYKFKENLQACLEAKRAASIFKSLPFEVTSMKDIQGLPCITDDVRDVIEEIIEEGESSRVKEILNDERYKAFKQFTSVFGVGVKTSEKWYRMGLRTVEEAKASKSLKLSKMQKAGFLYYEDLASGVSKEEADAVTLIVKETVRTVVPDALVTIAGGFRRGKPMGHDVDFLITNPGRPEDDELLHKVVDLLKKQGLLLYEDIRESTFVEGQLPSPKIDALDNHQKCFAILKLYQPKVDNSAVGTSKKSDMAEVKDWKAVRVDLVITPFEQFAYALLGWTGSGQFRRDLRRYAEEERDMLLDNHRLYDLKKGIFLSAGSEEEIFGHLGLDYLEPWERNA |
| >TdT-25  MHHHHHHGGSGHPEGNTPEKEVPSVIARHVSQYACQRKTTLNNYNKKFTDALEIMAENYKFKENLQACLEAKRAASVLKSLPFEVTSMKDIQGLPCITDDVRDVIEEIIEEGESSRVKEILNDERYKAFKQFTSVFGVGVKTSEKWYRMGLRTVEEAKASKSLKLSKMQKAGFLYYEDLASGVSKEEADAVTLIVKETVRTVSPDALVTIAGGFRRGKPMGHDVDLLITIPGRPEDDELLHKVVDLLKKQGLLLYEDIRESTFVEGQLPSPKIDALDNFQKCFAILKLYRPKVDNSAVGTSKKSDMAEVKDWKAVRVDLVITPFEQFAYALLGWTGSGQFRRDLRRYAEEERDMLLDNHRLYDLKKGIFLSAGSEEEIFGHLGLDYLEPWERNA |
| >TdT-26  MHHHHHHGGSGHPEGNTPEKEVPSVIARHVSQYACQRKTTLNNYNKKFTDALEIMAENYKFKENLQACLEAKRAASVLKSLPFEVTSMKDIQGLPCITDDVRDVIEEIIEEGESSRVKEILNDERYKAFKQFTSVFGVGVKTSEKWYRMGLRTVEEAKASKSLKLSKMQKAGFLYYEDLASGVSKEEADAVTLIVKETVRTVSPDALVTIAGGFRRGKPMGHDVDLLITIPGRPEDDELLHKVVDLLKKQGLLLYEDIRESTFVEGQLPSPKPDALDNHQKCFAILKLYRPKVDNSAVGTSKKSDMAEVKDWKAVRVDLVITPFEQFAYALLGWTGSGQFRRDLRRYAEEERDMLLDNHRLYDLKKGIFLSADSEEEIFGHLGLDYLEPWERNA |
| >TdT-27  MHHHHHHGGSGHPEGNTPEKEVPSVIARHVSQYACQRKTTLNNYNKKFTDALEIMAENYKFKENLQACLEAKRAASVLKSLPFEVTSMKDIQGLPCITDDVRDVIEEIIEEGESSRVKEILNDERYKAFKQFTSVFGVGVKTSEKWYRMGLRTVEEAKASKSLKLSKMQKAGFLYYEDLASGVSKEEADAVTLIVKETVRTVSPDALVTIAGGFRRGKPMGHDVDLLITIPGRPEDDELLHKVVDLLKKQGLLLYEDIRESTFVEGQLPNPKPDALDNHQKCFAILKLYRPKVDNSAVGTSKKSDMAEVKDWKAVRVDLVIAPFEQFAYALLGWTGSGQFRRDTRRYAEEERDMLLDNHRLYDLKKGIFLSADSEEEIFGHLDLDYLEPWERNA |
| >TdT-28  MHHHHHHGGSGHPEGNTPEKEVPSVIARHVSQYACQRKTTLNNYNKKFTDALEIMAENYKFKENLQACLEAKRAASVLKSLPFEVTSMKDIQGLPCITDDVRPVIEEIIEVGESSRVKEILNDERYKAFKQFTSVFGVGVKTSEKWYRMGLRTVEEAKASKSLKLSKMQKAGFLYYEDLASGVSKEEADAVTLIVKETVRTVSPDALVTIAGGFRRGKPMGHDVDLLITIPGRPTDDELLHKVVDLLKKQGLLLYEDIRESTFVEGQLPNPKPDALDNHQKCFAILKLYRPKVDNSAVGTSKKSDMAEVKDWKAVRVDLVVAPDEQFAYALLGWTGSGQFRRDTRRYAEEERDMLLDNHRLYDLKKGIFLSADSEEEIFGHLDLDYLEPWERNA |
| >TdT-29  MHHHHHHGGSGHPEGNTPEKEVPSVIARHVSQYACQRKTTLNNYNKKFTDALEIMAENYKFKENLQACLEAKRAASVLKSLPFEVTSMKDIQGLPCITDDVRPVIEEIIEVGESSRVKEILNDERYKAFKQFTSVFGVGVKTSEKWYRMGLRTREEAKASKSLKLNKMQKAGFLYYEDLASGVSKEEADAVTLIVKETVRTVSPDALVTIAGGFRRGKPMGHDVDLLITIPGRPTDDELLHKVVDLLKKQGLLLYEDIRESTFVEGQLPNPKPDALDNHQKCFAILKLYRPKVDNSAVGTSKKSDMAEVKDWKAVRVDLVVAPDEQFAYALLGWTGSGQFRRDTRRYAEEERDMLLDNHRLYDLKKGIFLSADSEEEIFGHLDLDYLEPWERNA |
| >TdT-30  MHHHHHHGGSGHPEGNTPEKEVPSVIARHVSPYACQRKTTLNNYNKKFTDALEIMAENYKFKENLQACLEARRAASVLKSLPFEVTSMKDIQGLPCITDDVRPVIEEIIEVGESSRVKEILNDERYKAFKQFTSVFGVGVKTSEKWYRMGLRTREEAKASKSLKLNKMQKAGFLYYEDLASGVSKEEADAVTLIVKETVRTVSPDALVTIAGGFRRGKPMGHDVDLLITIPGRPTDDELLHKVVDLLKKQGLLLYEDIRESTFVEGQLPNPKPDALDNHQKCFAILKLYRPKVDNSAVGTSKKSDMAEVKDWKAVRVDLVVAPDEQFAYALLGWTGSGMFRRDTRRYAEEERDMLLDNHGLYDLKKGIFLSADSEEEIFGHLDLDYLEPWERNA |
| >TdT-31  MHHHHHHGGSGHPEGNTPEKEVPSVIARHVSPYACQRKTTLNNYNKKFTDALEIMAENYKFKENLQACLEARRAASVLKSLPFEVTSMKDIQGLPCITDDVRPVIEEIIEVGESSRVKEILNDERYKAFKQFTSVFGVGVKTSEKWYRMGLRTREEAKASKSLKLNKMQKAGFLYYEDLASGVSKEEADAVLDIVKETVRTVSPDALVTIAGGFRRGKPMGHDVDLLITIPGRPTDDELLHKVVDLLKKQGLLLYEDIRESTFVEGQLPNPKPDALDNHQKCFAILKLYRPKVDNSAVGTSKKSDMAEVKDWKAVRVDLVVAPDEQFAYALLGWTGSGMFRRDTRRYAEEERDMLLDNHGLYDLKKGIFLSADSEEEIFGHLDLDYLEPWERNA |
| >TdT-32  MHHHHHHGGSGHPEGNTPEKEVPSVIARHVSPYACQRKTTLNNYNKKITDALEIMAENYKFKENLQACLEARRAASVLKSLPFEVTSMKDIQGLPCITDDVRPVIEEIIEVGESSRVKEILNDERYKAFKQFTSVFGVGVKTSEKWYRMGLRTREEAKASKSLKLNKMQKAGFLYYEDLASGVSKEEADAVLDIVKETVRTVSPDALVTIAGGFRRGKPMGHDVDLLITIPGRPTDDELLHKVVDLLKKQGLLLYGDIRERTFVEGQLPNPKPDALDNHQKCFAILKLYRPKVDNSAVGTSKKSDMAEVKDWKAVRVDLVVAPDEQFAYALLGWTGSGMFRRDTRRYAEEERDMLLDNHGLYDLKKGIFLSADSEEEIFGHLDLDYLEPWERNA |
| >TdT-33  MHHHHHHGGSGHPEGNTPEKEVPSVIARHVSPYACQRKTTLNNYNKKITDALEIMAENYKFKENLQARLEARRAASVLKSLPFEVTSMKDIQGLPCITDDVRPVIEEIIEVGESSRVKEILNDERYKAFKQFTSVFGVGVKTSEKWYRMGLRTREEAKASKSLKLNKMQKAGFLYYEDLASGVSKEEADAVLDIVKETVRTVSPDALVTIAGGFRRGKPMGHDVDLLITIPGRPTDDELLHKVVDLLKKQGLLLYGDIRERTFVEGQLPNPKPDALDNHQKCFAILKLYRPKVDNSAVGTSKKSDMAEVKDWKAVRVDLVVAPDEQFAYALLGWTGSGMFRRDTRRYAEEERDMLLDNHGLYDLKKGIFLSADSEEEIFGHLDLDYLEPWERNA |

**Supplementary Table 10. TdT variant MSA.** Residues changed with respect to **TdT-1** are highlighted.


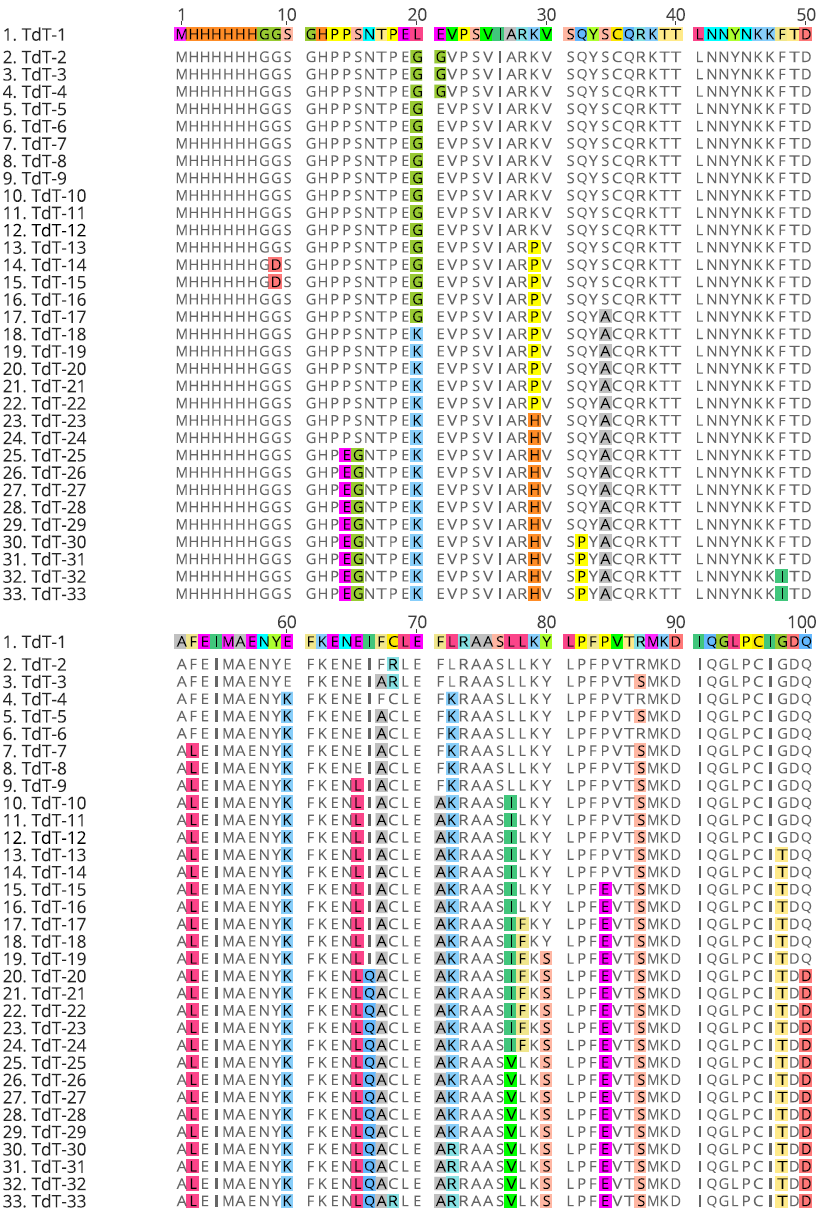


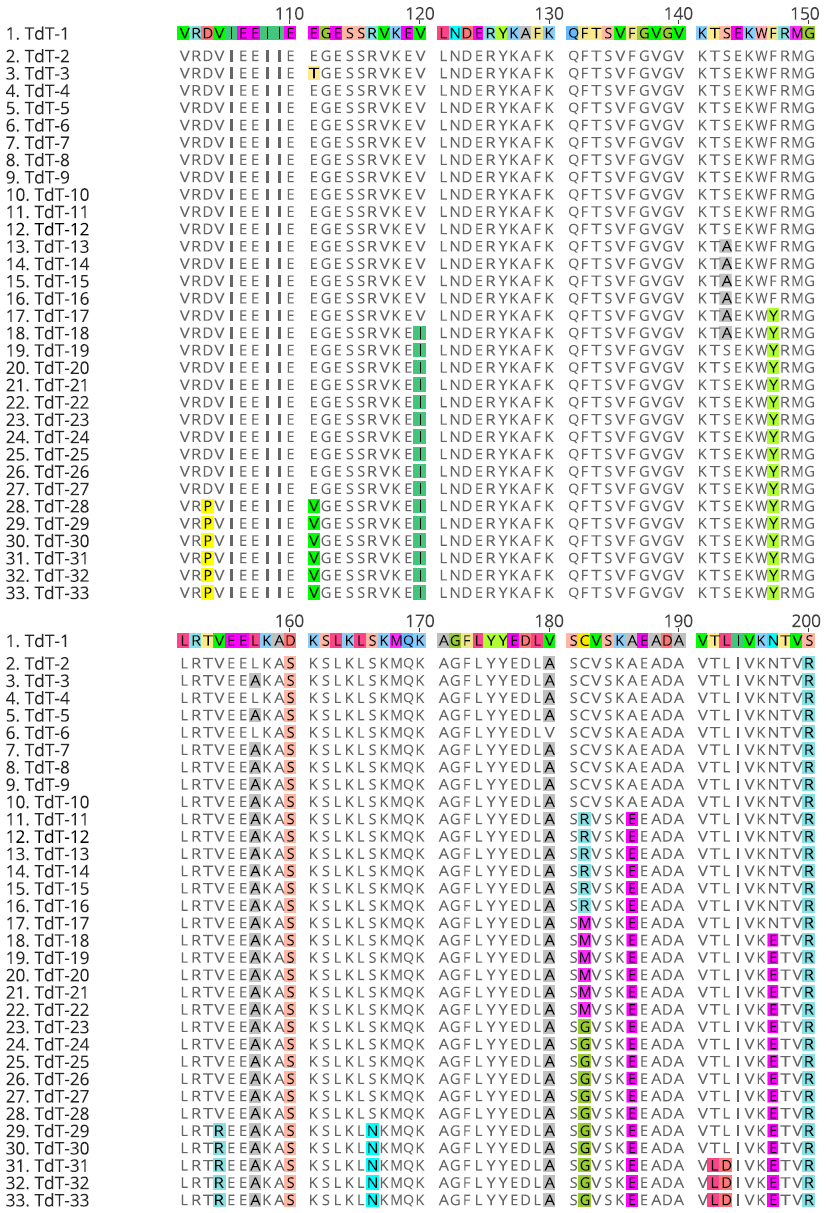


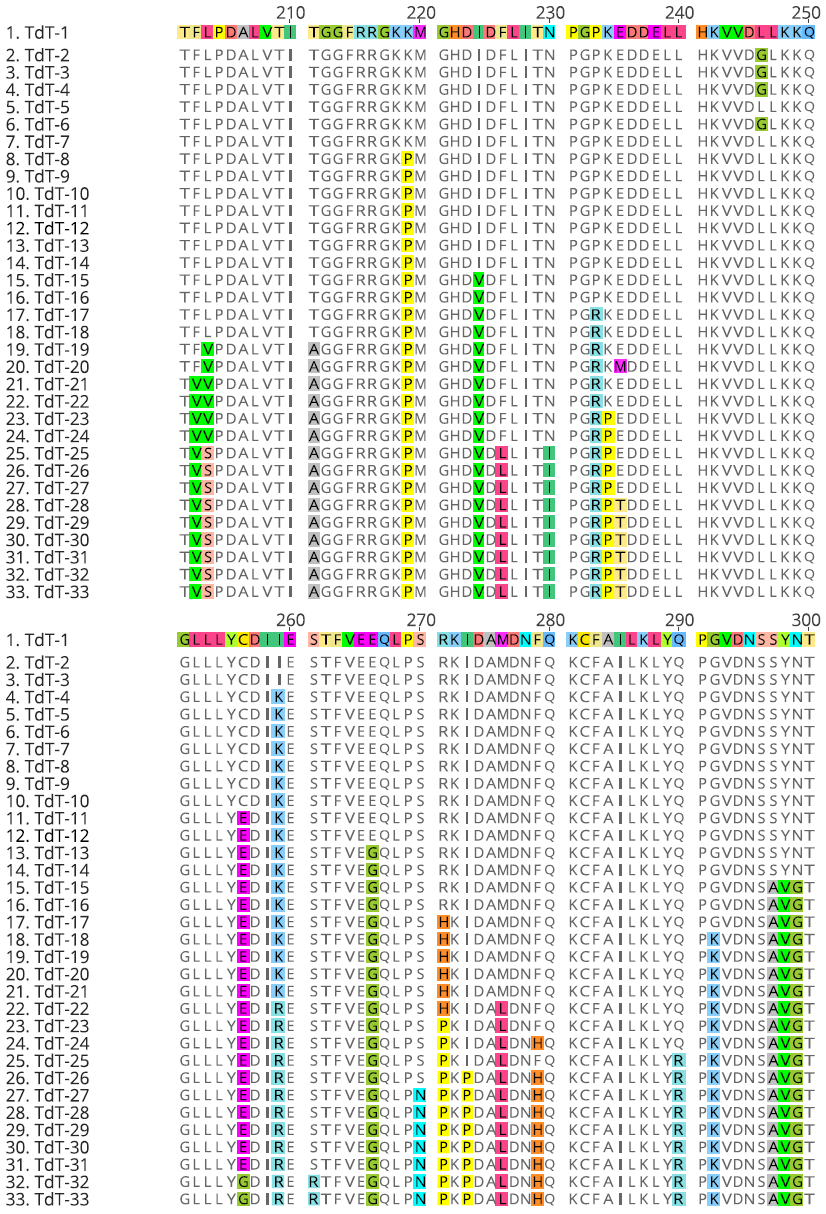


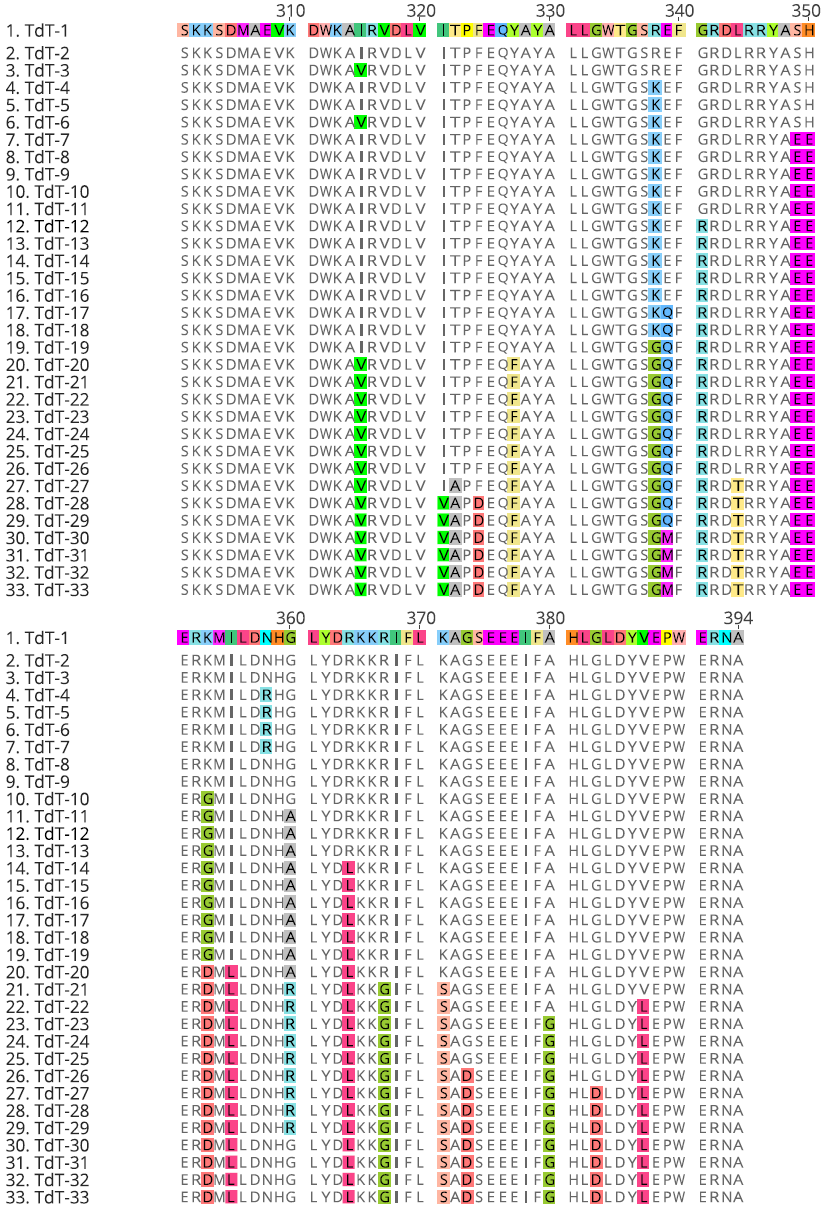


**Supplementary Table 11. Directed evolution round summary.**

| **Rd** | **Rd parent** | **Protein preparation conditions** | **Protein preincubation** | **Oligo acceptor** | **Nucleotide substrate** | **Reaction volume** | **Lysate dilution** | **Reaction Temp, time** | **Analysis method** | **New parent selected** | **mutations  rel. to parent** | **Main factor for variant selection** |
| --- | --- | --- | --- | --- | --- | --- | --- | --- | --- | --- | --- | --- |
| 1 | TdT-1 | Lysozyme-lysed clarified lysate | 49 °C, 1h | TAATT, 2 μM | ddTTP,  100 μM | 20 μL | N/A | 49 °C, 60min | LCMS | TdT-2 | L20G; E21G; C68R; D160S; V180A; S200R; L246G; | Stability/ solubility |
| 2 | TdT-2 | Lysozyme-lysed clarified lysate | 49 °C, 1h | TAATT, 2 μM | ddTTP,  100 μM | 20 μL | N/A | 49 °C, 60min | LCMS | TdT-3 | F67A; R87S; E111T; L157A; I315V; | Stability/ solutbility |
| 3 | TdT-2 | Lysozyme and B-Per lysis, HTP-purified protein | 25 °C, 1h | TTTTTTTATC, 4 μM | 3'PO_4_-dCTP, 200 μM | 20 μL | N/A | 40 °C, 180min | LCMS | TdT-4 | E60K; R68C; L72K; I259K; R338K; N358R; | Activity on 3’-phosphate |
| 4 | TdT-4 | Lysozyme and B-Per lysis, HTP-purified protein | 25 °C, 1h | TTTTTTTATC, 4 uM | 3'PO_4_-dCTP, 200 μM | 20 μL | N/A | 40 °C, 180min | LCMS | TdT-5 | G21E; F67A; R87S; L157A; G246L; | Activity on 3’-phosphate |
| 5 | TdT-4 | Lysozyme and B-Per lysis, HTP-purified protein | 25 °C, 1h | TTTTTTTATC, 4 μM | 3'PO_4_-dCTP, 200 μM | 20 μL | N/A | 40 °C, 180min | LCMS | TdT-6 | G21E; F67A; A180V; I315V; | Activity on 3’-phosphate |
| 6 | TdT-5 | Lysozyme and B-Per lysis, HTP-purified protein | 25 °C, 1h | TTTTTTTATC, 4 μM | 3'PO_4_-dCTP, 150 μM | 20 μL | N/A | 40 °C, 90min | LCMS | TdT-7 | F52L; S349E; H350E; | Activity on 3’-phosphate |
| 7 | TdT-7 | Lysozyme and B-Per lysis, HTP-purified protein | 25 °C, 1h | TTTTTTTATC, 4 μM | 3'PO_4_-dTTP, 50 μM | 20 μL | N/A | 42 °C, 60min | LCMS | TdT-8 | K219P; R358N; | Specific activity |
| 8 | TdT-8 | Lysozyme and B-Per lysis, HTP-purified protein | 25 °C, 1h | TTTTTTTATC, 4 μM | 3'PO_4_-dTTP, 50 μM | 20 μL | N/A | 42 °C, 60min | LCMS | TdT-9 | E65L | Specific activity |
| 9 | TdT-9 | Lysozyme-lysed clarified lysate | 25 °C, 1h | TTTTTTTATC, 4 μM | 3'PO_4_-dCTP, 50 μM | 20 μL | N/A | 46 °C, 60min | LCMS | TdT-10 | F71A; L77I; K353G; | Specific activity |
| 10 | TdT-10 | Lysozyme-lysed clarified lysate | 46 °C, 1h | TTTTTTTATC, 4 μM | 3'PO_4_-dGTP, 50 μM | 20 μL | N/A | 50 °C, 30min | LCMS | TdT-11 | C182R; A186E; C256E; G360A; | Specific activity/ stability |
| 11 | TdT-11 | Lysozyme-lysed clarified lysate | 44 °C, 1h | TTTTTTTATC, 4 μM | 3'PO_4_-dCTP, 50 μM | 20 μL | N/A | 46 °C, 15min | LCMS | TdT-12 | G341R; | Specific activity/ stability |
| 12 | TdT-12 | Lysozyme-lysed clarified lysate | 50 °C, 1h | TTTTTTTATC, 4 μM | 3'PO_4_-dGTP, 50 μM | 20 μL | N/A | 50 °C, 15min | LCMS | TdT-13 | K29P; G98T; S143A; E266G; | Specific activity/ stability |
| 13 | TdT-13 | Lysozyme-lysed clarified lysate | 53 °C, 1h | TTTTTTTATC, 4 μM | 3'PO_4_-dGTP, 50 μM | 20 μL | N/A | 53 °C, 15min | LCMS | TdT-14 | G9D; R364L; | Specific activity/ stability |
| 14 | TdT-14 | Lysozyme-lysed clarified lysate | 55 °C, 1h | TTTTTTTATC, 4 μM | 3’PO_4_-dGTP, 50 μM | 36 μL | N/A | 55 °C, 15min | RapidFire SPE-MS/MS | TdT-15 | P84E; I224V; S297A; Y298V; N299G; | Specific activity/ stability |
| 15 | TdT-15 | Lysozyme-lysed clarified lysate | 52 °C, 1h | TTTTTTTATC, 4 μM | 3’PO_4_-dGTP, 50 μM | 36 μL | N/A | 52 °C, 15min | RapidFire SPE-MS/MS | TdT-16 | D9G | Specific activity |
| 16 | TdT-16 | Lysozyme-lysed clarified lysate | 58 °C, 1h | TTTTTTTATC, 4 μM | 3’PO_4_-dTTP, 50 μM | 36 μL | N/A | 58 °C, 15min | RapidFire SPE-MS/MS | TdT-17 | S34A; L78F; F147Y; R182M; P233R; R271H; E339Q; | Specific activity/ stability |
| 17 | TdT-17 | Lysozyme-lysed clarified lysate | 60 °C, 1h | TTTTTTTATC, 4 μM | 3’PO_4_-dCTP, 50 μM | 36 μL | N/A | 60 °C, 15min | RapidFire SPE-MS/MS | TdT-18 | G20K; V120I; N197E; G292K; | Stability |
| 18 | TdT-18 | Lysozyme-lysed clarified lysate | 56 °C, 1h | TTTTTTTATG, 4 μM | 3’PO_4_-dATP, 50 μM | 36 μL | 4x | 56 °C, 15min | RapidFire SPE-MS/MS | TdT-19 | Y80S; A143S; L203V; T211A; K338G; | Specific activity |
| 19 | TdT-19 | Lysozyme-lysed clarified lysate | 62 °C, 1h | TTTTTTTATC, 4 μM | 3’PO_4_-dGTP, 50 μM | 36 μL | N/A | 62 °C, 15min | RapidFire SPE-MS/MS | TdT-20 | I66Q; Q100D; E235M; I315V; Y327F; G353D; I355L; | Stability |
| 20 | TdT-20 | Lysozyme-lysed clarified lysate | 56 °C, 1h | TTTTTTTACA, 4 μM | 3’PO_4_-dTTP, 50 μM | 36 μL | 4x | 56 °C, 15min | RapidFire SPE-MS/MS | TdT-21 | F202V; M235E; A360R; R367G; K371S; | Specific activity |
| 21 | TdT-21 | Lysozyme-lysed clarified lysate | 60 °C, 1h | TTTTTTTATG, 4 μM | 3’PO_4_-dATP, 50 μM | 36 μL | 8x | 60 °C, 15min | RapidFire SPE-MS/MS | TdT-22 | K259R; M276L; V387L; | Specific activity |
| 22 | TdT-22 | Lysozyme-lysed clarified lysate | 60 °C, 1h | TTTTTTTCGG, 4 μM | 3’PO_4_-dGTP, 50 μM | 36 μL | 4x | 60 °C, 15min | RapidFire SPE-MS/MS | TdT-23 | P29H; M182G; K234P; H271P; A380G; | Specific activity |
| 23 | TdT-23 | Shake-flask expressed and purified protein | no prein °Cubation | 5'-6-FAM-TTTTTTTTTTTTTTTTTATC or  5'-6-FAM-TTTTTTTTTTTTTTTTTGTT,  1 μM | 3'PO_4_-dGTP, 25 μM | 20 μL | N/A; 1 μM enzyme | 60 °C, 1.5min | Capillary electrophoresis | TdT-24 | F279H; | Specific activity |
| 24 | TdT-24 | Lysozyme-lysed clarified lysate | 60 °C, 1h | TTTTTTTCGA, 4 μM | 3’PO_4_-dTTP, 50 μM | 36 μL | 4x | 60 °C, 15min | RapidFire SPE-MS/MS | TdT-25 | P14E; S15G; I77V; F78L; V203S; F226L; N230I; Q290R; | Specific activity |
| 25 | TdT-23 | Lysozyme-lysed clarified lysate | 60 °C, 1h | TTTTTTTCGG, 4 μM | 3’PO_4_-dGTP, 50 μM | 36 μL | 16x | 60 °C, 15min | RapidFire SPE-MS/MS | TdT-26 | I273P; F279H; G373D; | Specific activity |
| 26 | TdT-26 | Lysozyme-lysed clarified lysate | 65 °C, 1h | TTTTTTTATC, 4 μM | 3’PO_4_-dATP, 50 μM | 36 μL | 16x | 65 °C, 15min | RapidFire SPE-MS/MS | TdT-27 | S270N; T322A; L344T; G383D; | Specific activity |
| 27 | TdT-27 | Lysozyme-lysed clarified lysate | 65 °C, 1h | TTTTTTTATC, 2 μM | 3’PO_4_-dATP, 25 μM | 36 μL | 64x | 65 °C, 10min | RapidFire SPE-MS/MS | TdT-28 | D103P; E111V; E235T; I321V; F324D; | Stability |
| 28 | TdT-28 | Lysozyme-lysed clarified lysate | 60 °C, 1h | TTTTTTTGTT, 2 μM | 3’PO_4_-dTTP, 25 μM | 36 μL | 32x | 60 °C, 5min | RapidFire SPE-MS/MS | TdT-29 | V154R; S166N; | Specific activity |
| 29 | TdT-29 | Lysozyme-lysed clarified lysate | 60 °C, 1h | TTTTTTTGTT, 2 μM | 3’PO_4_-dTTP, 15 μM | 36 μL | 32x | 60 °C, 5min | RapidFire SPE-MS/MS | TdT-30 | Q32P; K72R; Q339M; R360G; | Specific activity |
| 30 | TdT-30 | Lysozyme-lysed clarified lysate | 60 °C, 1h | TTTTTTTCGA, 2 μM | 3’PO_4_-dTTP, 15 μM | 36 μL | 32x | 60 °C, 5min | RapidFire SPE-MS/MS | TdT-31 | T192L; L193D; | Specific activity |
| 31 | TdT-31 | Lysozyme-lysed clarified lysate | 70 °C, 1h | TTTTTTTATC, 2 μM | 3’PO_4_-dATP, 10 μM | 36 μL | 16x | 70 °C, 2min | RapidFire SPE-MS/MS | TdT-32 | F48I; E256G; S261R; | Stability |
| 32 | TdT-32 | Lysozyme-lysed clarified lysate | 60 °C, 1h | TTTTTTTGGA, 1 μM | 3’PO_4_-dTTP, 5 μM | 36 μL | 8x | 60 °C, 2min | RapidFire SPE-MS/MS | TdT-33 | C68R | Specific activity |
